# Incorporating genetic selection into individual‐based models of malaria and other infectious diseases

**DOI:** 10.1111/eva.13077

**Published:** 2020-08-11

**Authors:** Ian M. Hastings, Diggory Hardy, Katherine Kay, Raman Sharma

**Affiliations:** ^1^ Liverpool School of Tropical Medicine Liverpool UK; ^2^ Swiss Tropical and Public Health Institute Basel Switzerland; ^3^ University of Basel Basel Switzerland; ^4^ Metrum Research Group Tariffville CT USA

**Keywords:** computer simulation, diagnosis, drug resistance, genetics, population, malaria, mass drug administration, mutation, vaccines

## Abstract

**Introduction:**

Control strategies for human infections are often investigated using individual‐based models (IBMs) to quantify their impact in terms of mortality, morbidity and impact on transmission. Genetic selection can be incorporated into the IBMs to track the spread of mutations whose origin and spread are driven by the intervention and which subsequently undermine the control strategy; typical examples are mutations which encode drug resistance or diagnosis‐ or vaccine‐escape phenotypes.

**Methods and results:**

We simulated the spread of malaria drug resistance using the IBM OpenMalaria to investigate how the finite sizes of IBMs require strategies to optimally incorporate genetic selection. We make four recommendations. Firstly, calculate and report the selection coefficients, *s*, of the advantageous allele as the key genetic parameter. Secondly, use these values of “*s*” to calculate the wait time until a mutation successfully establishes itself in the pathogen population. Thirdly, identify the inherent limits of the IBM to robustly estimate small selection coefficients. Fourthly, optimize computational efficacy: when “*s*” is small, fewer replicates of larger IBMs may be more efficient than a larger number of replicates of smaller size.

**Discussion:**

The OpenMalaria IBM of malaria was an exemplar and the same principles apply to IBMs of other diseases.

## 1 INTRODUCTION

Advances in computational power over the last 20 years have allowed sophisticated, individual‐based models (IBMs) of infectious diseases to be developed and applied to important human and animal disease. These are particularly valuable in diseases with complex transmission through vector species or which have complex clinical aetiology. IBMs allow considerably more realism to be added to the simple compartment models (such as the Ross–Macdonald approach for vector‐borne diseases or the more general susceptible–infected–recovered models) which are usually the first analyses used to investigate disease epidemiology. The next challenge for these IBMs is to incorporate genetic selection into the disease epidemiology. Most pathogens readily evolve in response to human interventions. For example, drug resistance almost inevitably evolves in response to drug deployment, and mutations arise that change the antigenic profile of the molecules detected by molecular diagnosis, making the pathogens invisible to diagnosis. Incorporating this evolution into IBMs is not straightforward, and this manuscript discusses how it may best be achieved in terms of accurately quantifying the selection process in a computationally efficient manner.

Our personal expertise lies in malaria for which there are several IBMs as recently, and comprehensively, reviewed by Smith et al. (2018). Several IBM models have been supported by the Bill and Melinda Gates Foundation to investigate interventions to control/eliminate malaria, and the consensus exercises co‐ordinated by these groups have been influential in evaluating the potential impact of intervention such as partially effective vaccines (e.g. Penny et al., 2016), mass drug administration programmes (e.g. Brady et al., 2017) and the impact of new diagnostic tools (e.g. Slater et al., 2015). These publications focused on the relatively short‐term impact of interventions but historically malaria populations evolve and adapt to the challenges posed by such interventions. The obvious examples are vaccine‐based interventions driving vaccine‐insensitive variants (e.g. Genton et al., 2002; Neafsey et al., 2015; discussion in Plowe, 2015), drug‐based interventions driving drug resistance (e.g. figure 3 of Blasco, Leroy, & Fidock, 2017) and deployment of rapid diagnostic tests (RDTs) driving mutations that prevent the infection being detected and diagnosed (such as *hrp2*‐deletion mutants; Verma, Bharti, & Das, 2018; World Health Organization, 2017). The basic dynamics of spread of such mutations can be obtained from standard population genetics methodology (e.g. Curtis & Otoo, 1986; Dye & Williams, 1997; Hastings, 1997 and later). However, the simplicity of these methodologies means the analyses must focus on malaria genetics and often largely ignore the epidemiological and clinical frameworks within which these selection processes operate. Consequently, there is increasing interest in incorporating genetic selection into the IBMs of malaria. The use of IBMs makes the selective background far more realistic and can incorporate factors such as heterogeneity in mosquito biting (e.g. Guelbéogo et al., 2018), the effectiveness of diagnosis (e.g. Slater et al., 2015), the effect of human acquired immunity (e.g. Crompton et al., 2014), the impact of superinfection, local patterns of clinical treatment (e.g. Nkumama, O'Meara, & Osier, 2017), among other factors. IBMs can also incorporate biological uncertainty by comparing results obtained by including/excluding the unknown effect, for example whether an infective inoculation consists of a single genetic entity, or a range of genetically related pathogens (e.g. Nkhoma et al., 2012, 2020), the impact of immunity and of local genetic structure on diversity (e.g. Chapter 10 of Frank, 2002), the presence of fitness costs associated with the mutation (e.g. Melnyk, Wong, & Kassen, 2015) and so on. Finally, we assumed resistance is encoded by a single gene so we can ignore the effects of sexual recombination.

Population genetic theory has been developed and refined over the last 100 years and hence largely in the absence of computer infrastructure. Consequently, there is a large amount of basic theory that can be combined with large‐scale IBMs of disease transmission to make selection more transparent, more computationally efficient and, importantly, to make the results and outputs comparable across simulation platforms. The aim of this paper was to describe how this may be achieved. The significant differences between the two approaches are that simple populations genetic theory usually assumes infinite population sizes (so that random fluctuations in allele frequency are absent) whereas IBMs track finite population sizes. There are two related effects that occur in finite populations that affect how we bring genetic selection into IBMs and these need to be understood before we describe the integration of population genetics into IBMs. Both effects arise because IBMs track numbers of each genotype of parasites (from which frequencies are extracted):

*Stochastic fluctuation may result in “genetic extinction” when small numbers of one allele type are present*. This effect emphasizes the difference between the expected change in the number of infections carrying the allele and the actual change. Suppose there are 10 infections carrying the advantageous allele (e.g. drug resistance) in the IBM and the selection coefficient (see Section 2.1) acting on the allele is 0.05, that is the allele frequency is expected to increase by 5% per malaria generation which is roughly in line with field estimates of the selection coefficient (see Recommendation #1 in Section 3). The expected number of resistant infections next generation is 10*1.05 = 10.5 but it is obviously impossible to leave exactly 10.5 infections: the number of resistant infections must come from a distribution, that is 0,1,2,3,4,5, (i.e. fractions of infections are not possible) and variation in numbers of transmission is typically large. Importantly, there is a small, but finite, probability of leaving zero infections next generation; in the case “genetic extinction” has occurred, that is the allele has been lost from the populations. For a real example of this effect, see later discussion of Figure 5a). The risk of genetic extinction is greatest when there are small numbers of one type of allele. This is most notable for new mutations, which are by definition, present at a single copy when they first arise; in that case, even with a selective advantage of 5%, the mutation will be lost by chance between 90% and 99% of the time depending on the level of heterogeneity in transmission (see discussion in box 2 of Hastings, 2004). Hence, it is highly desirable to avoid low numbers of infections with any allelic type in the IBM as they may go genetically extinct purely by chance, even if that allele is advantageous over the longer term.
*Genetic drift* is similar to the stochastic change described above but occurs at higher numbers. The risk of random genetic extinction has largely passed when there are large numbers of the advantageous allele, but random fluctuations may obscure the underlying selection. Suppose there are 1,000 resistant infections with selective advantage of 5%, then on average there will be 1,000*1.05 = 1,050 next generation. But, the stochastic variation described above will still occur and cause chance variation around this expected number. This is termed genetic drift and introduces “noise” that causes variation in the spread of the advantageous allele. A critical point is that at low selection coefficients, the drift can completely obscure the dynamics of spread: in effect the “signal” (selection pressure favouring the alleles) is lost in the “noise” (genetic drift). Intuitively, and correctly, the effects of drift become more pronounced at small population sizes, that is the smaller the number of infections tracked in the IBM, the larger the impact of drift. This is a well‐known phenomenon in population genetics which, for our purposes, sets a limit on the sensitivity of the IBMs to track selection. That is, for any given size of IBM there comes a point when the selection coefficient becomes so small that it is obscured by drift and the IBM can no longer effectively track the genetics. The impact of this effect is discussed in detail later in Section 2.3

A final key genetic concept is that of effective population size, *Ne*, and the “census” population size, *N*. The latter is simply the number of all malaria clones in the population (i.e. of all allelic types) which we will take as the number of infected humans present in the IBMs (ignoring, for convenience, the fact that some infections may be superinfections, i.e. consist of several genetically distinct *P. falciparum* clones). In other words, it is the number of *infected* humans in the simulation, not the total number of humans tracked, that predominantly determines the population genetic properties of the simulation. Population genetic theory used to investigate finite population sizes has been developed for idealized, paradigm population that is assumed to have a constant census size *N* and whose members are assumed to have equal reproductive potential. Most real populations, including most infectious disease species, do not fit this paradigm, which led to the concept of an effective population size which is the size of the paradigm population that would have equivalent properties of that of the census size *N* (see Kliman, Sheehy, & Schultz, 2008 for an introduction to *N*, *Ne* and its impact on genetic drift). Importantly, *Ne* in natural populations is invariably much smaller than *N*. Notably, there is considerable heterogeneity in the reproductive success of many infections depending on host factors such as the level of host immunity, the number of secondary contacts made by that person (if the disease is directly transmitted) or how often that person is bitten (if the infection is indirectly transmitted by disease vectors such as ticks or mosquitoes). *Ne* also falls as pathogen populations “bottleneck” due to seasonal patterns of transmission (a common phenomenon associated with mosquito‐transmitted disease), and large‐scale control programmes (such as bed net distribution programs to control malaria) may cause significant reductions in pathogen census population sizes. These factors will substantially reduce *Ne* with important consequences for genetic drift which, as we show later in Figure 6, limits the sensitivity of IBMs to track selection of advantageous alleles with low selection coefficients. The important point is that the number of infections being tracked, *N*, may appear high, but *Ne* may be considerably lower which has a large impact on the IBMs' ability to quantify the spread of advantageous mutations.

The purpose of this manuscript was to describe how these effects of finite population sizes need to be clearly recognized when incorporating genetic selection into IBMs and describe and discuss how their impact may be mitigated.

## 2 METHODS AND RESULTS

We use the individual‐based malaria simulation package OpenMalaria (e.g. Smith, Killeen, et al., 2006; Smith, Maire, et al., 2006; Smith et al., 2008; http://github.com/SwissTPH/openmalaria/wiki) to simulate the spread of drug resistance. This is a highly sophisticated IBM of malaria transmission that incorporates factors such as the acquisition of human immunity against malaria infection, local treatment practices, the level of mosquito transmission and has been widely used to investigate many aspects of malaria transmission and control. The consequence of this sophistication is that it is highly computationally intensive, so makes an ideal test platform to develop computationally efficient methods of incorporating genetic selection. Details of our assumptions and calibrations for the OpenMalaria simulations in this work are given in Supporting Information but, in summary, we run the simulation model for a warm‐up period of 99 years, and then over an additional 10‐year burn‐in period before introducing the advantageous allele. OpenMalaria outputs the number of inoculations of each allelic type every 5 days as a cumulative total over that period. We extract the proportion of inoculations carrying the advantageous allele from each 5‐day time point to monitor the spread of the advantageous allele and it is these data that enter the regression to estimate the selection coefficient. The number of humans to be tracked in OpenMalaria is user‐specified (we track 10,000 unless otherwise indicated), and we can vary the user‐defined entomological inoculation rate (EIR; the mean number of infective bites per adult human per year) to vary the prevalence of malaria infection; by default, we simulate a prevalence of 15% averaged over all ages based on diagnosis by microscopy (diagnosis in OpenMalaria is probabilistic with a 50% chance of a positive diagnosis when parasite density reaches 20 parasite/μL).

In these simulations, we assume the “advantageous allele” is one encoding drug resistance; the same principle applies to all advantageous alleles, but alleles encoding drug resistance allow us to easily alter their selective advantage simply by altering their level of resistance to the drug being deployed. In the following examples, the drug is assumed to be dihydroartemisinin + piperaquine (DHA + PPQ) which is a widely used front‐line antimalarial drug (Annex 3B of World Health Organization (2019) noting this does not capture its widespread use through the private sector (e.g. Kioko et al., 2016), particularly in SE Asia where drug resistance has historically first emerged). We simulate different selection intensities driving the advantageous allele by varying the resistance level (IC50) to PPQ; increasing the IC50 value encoded by the advantageous allele increases the level of drug resistance and hence defines more highly advantageous mutations (Table 1). This strategy allows us to investigate the range of selection coefficients that covers “typical” values for drug resistance selection which has been estimated at ~0.02 to ~0.12 (see Recommendation #1 of Section 3). Results obtained for IC50 shifts of 1.1× and 1.4× IC_50_ fold are used as illustrative examples (except in part 2.3) as they represent selection coefficients of ~0.02 and ~0.06, respectively, using our default prevalence of 15%. Values higher than this gave largely predictable, deterministic results, and IC_50_ shifts in between these values of 1.1× and 1.4× were found to behave in an intermediate fashion that therefore added little to the overall picture.

**TABLE 1:** How increasing the drug resistance level of the advantageous allele increases its selection coefficient, *s*, and how the magnitude of *s* may vary according to infection epidemiology. Drug resistance level is defined as the fold increase in IC50 to the antimalarial drug piperaquine compared to the wild‐type allele. Malaria treatment and epidemiology are as described as in the main text and the simulations track 100,000 humans. The left column reports selection coefficients obtained when malaria prevalence was 15%, while the right column reports selection coefficients obtained when prevalence was 1.5%; all estimates started from an advantageous allele frequency of 10%

Drug resistance level	Selection coefficient (±*SE*) 15% prevalence	Selection coefficient (±*SE*) 1.5% prevalence
×1.0125	0.002 ± 0.0005	0.0001 ± 0.002
×1.025	0.004 ± 0.0004	−0.0004 ± 0.002
×1.05	0.008 ± 0.0004	0.002 ± 0.002
×1.1	0.016 ± 0.0004	0.004 ± 0.002
×1.2	0.030 ± 0.0004	0.004 ± 0.002
×1.4	0.056 ± 0.0004	0.005 ± 0.002
×2.4	0.13 ± 0.0004	0.017 ± 0.001

Boxplots were produced using the boxplot() function in the R base graphics package. The grey boxes show the interquartile range (IQR), that is contain the second and third quartiles of the data with the red horizontal line representing the median. Outliers are defined as observations lying outside of the range Q2‐1.5*IQR to Q3 + 1.5*IQR where Q2 is the lower boundary of quartile 2 and Q3 is upper boundary of quartile 3. The whiskers then denote the lower limit of quartile 1 and upper limit of quartile 4, excluding these outliers which are represented by circles.

### 2.1 Methods used to measure and report the dynamics of spread

**FIGURE 1:**
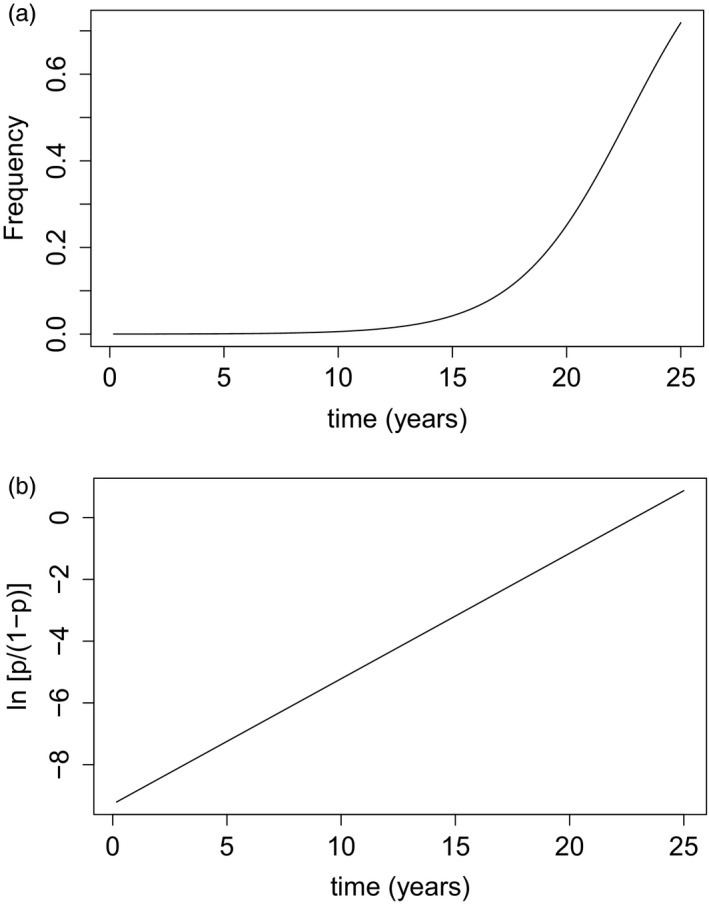
The spread of an advantageous allele in haploid organisms such as bacteria (Dykhuizen & Hartl, 1981) or malaria (Anderson & Roper, 2005); this example illustrates dynamics for a selective advantage of *s* = 0.07. Panel (a) shows spread on an arithmetic scale starting from a frequency of 0.0001. Much of the timescale occurs at frequencies that are essentially undetectable before becoming detectable in surveys of a reasonable size. Panel (b) shows the same data plotted as its logit, ln[*p*/(1 − *p*)], where *p* is the frequency of the advantageous allele and (1 − *p*) is the frequency of the wild‐type allele

Figure 1a shows the spread of an advantageous allele in a haploid organism. Most selection processes start from low frequency so most of the timescale taken to reach operationally significant frequencies occurs at low frequencies. Tracking the entire selection period is both computationally intensive and will also suffer from stochastic fluctuations in low allele number as described above. Fortunately, bacteria and malaria are haploid (we know of no selection that occurs in malaria's brief diploid phase in mosquito oocysts) so dominance between the two alleles is not an issue and the spread can be linearized as shown on Figure 1b. This offers a methodological alternative to measuring spread over a long duration at low frequency: it suggests that, providing selection pressure does not alter as a consequence of allele frequency (an important assumption, see Recommendation #4 of Section 3) that spread can be measured over a short period of time in the region of 10–15 malaria generations or around 2–3 years (which is computationally convenient), quantified at relatively high frequencies (minimizing the impact of stochastic changes in allele frequency) and extrapolated to the whole selection process.

We argue that this extrapolation is best achieved by estimating the selection coefficient, usually denoted “*s,”* to quantify the rate of spread. This parameter describes the relative “fitness” of the mutant as 1 + *s* compared to the fitness of the wild type which has fitness 1.0 and is conventionally reported in units of a single malaria generation (see below). Hence, at low frequencies, the mutant spreads at a rate *s* per generation (so if *s* = 0.05, then it increases by 5% per generation). Selection coefficient can be easily measured independently of allele frequency, as the slope of ln[*p*/(1 − *p*)] over time where *p* is the frequency of the advantageous allele (Figure 1b). Computer simulations generally simulate chronological time, for instance 1‐day timesteps, so we need to convert the slope of ln[*p*/(1 − *p*)] from chronological time to generations. In the current example, this requires an estimate of the duration of a malaria generation. In previous work, we have assumed five generations per year (Hastings & Donnelly, 2005) although other authors used different values, for example Anderson and Roper (2005) assumed six generations per year. Here, we will assume six generations per year meaning that each generation lasts 365/6≈60 days so the slope of ln[*p*/(1 − *p*)] estimated on a timescale of days needs to be multiplied by 60 to obtain the selection coefficient. Finally, note that *s* can be negative if the allele is actively being removed from the population (e.g. by natural selection if the allele is no longer being selected and has a fitness cost) or if the allele frequency decreases due to chance fluctuations (genetic drift); the principles described above apply equally to this situation, that is its magnitude is measured as ln[*p*/(1 − *p*)] over time but it will have a negative value (see, e.g., figure 3 of Anderson & Roper, 2005).

The dynamics shown on Figure 1 are those predicted by elementary population genetic theory, that is, constant selection pressure in an idealized population of infinite size. Individual‐based simulations do not fit this paradigm as selection coefficients may vary over time, most plausibly as a function of allele frequency (see Recommendation #4 in Section 3), and IBMs, by definition, do not track infinitely large populations. Many models and IBMs have a “burn‐in” period to allow infection epidemiology and thus population immunity to stabilize in the presence of a force of infection (FOI) prior to introducing interventions or the advantageous allele and tracking its spread. This “burn‐in” period forces researchers to make three key decisions when estimating selection coefficients after introduction of the advantageous allele, that is what should be the starting frequency of the advantageous allele when introduced into the IBM, when should measurement of ln[*p*(1 − *p*)] start and how long should measurement last? We illustrate the trade‐offs inherent in making these decisions.

**FIGURE 2:**
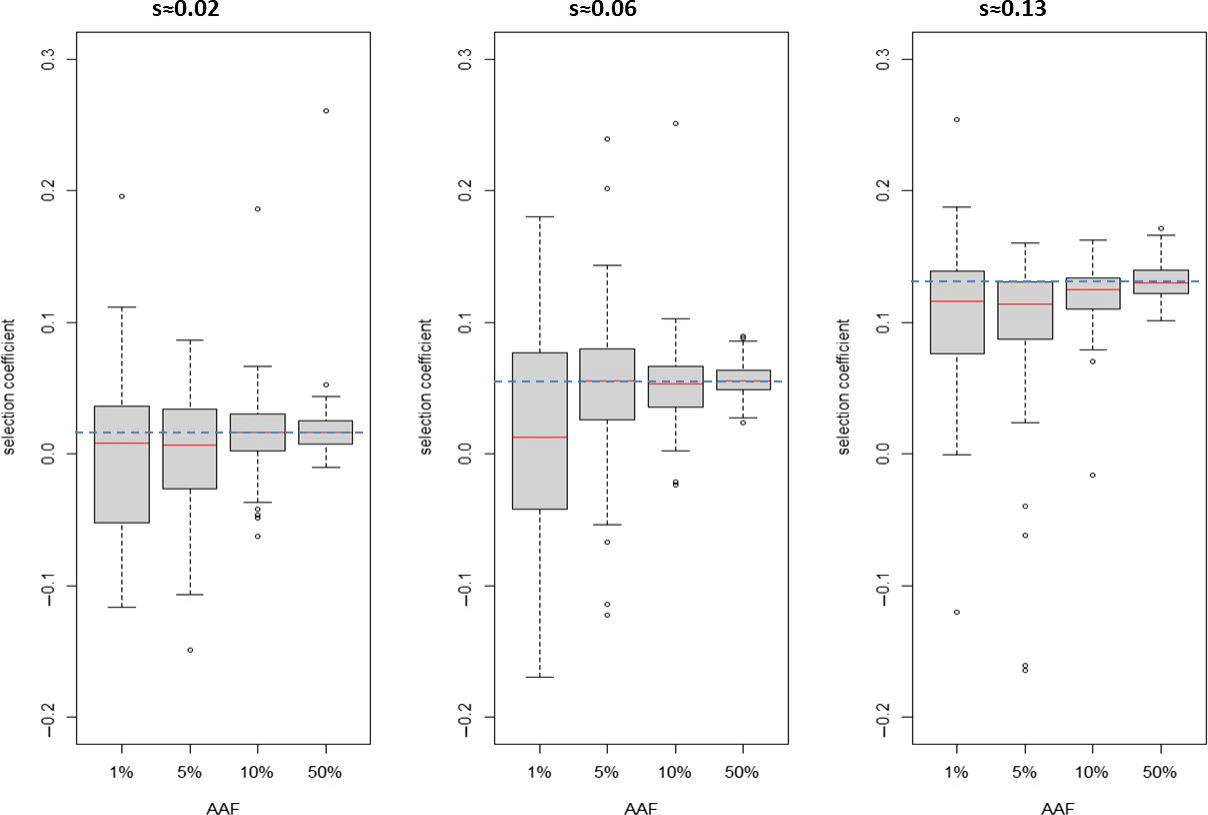
How starting advantageous allele frequency (AAF) affects estimation of selection coefficient, *s*. The boxplots each summarize 98–100 estimates* of selection coefficients obtained by simulating 10,000 humans with malaria prevalence of 15% using a regression window that starts 60 days after introduction of the advantageous allele and lasts the next 720 days (assuming AAF stay within bounds, details in main text). Left column: the advantageous allele encodes a 1.1‐fold increase in drug resistance (*s*≈0.02). Centre column: the advantageous allele encodes a 1.4‐fold increase in drug resistance (*s*≈0.06). Right column: the advantageous allele encodes a 2.4‐fold increase in drug resistance (*s*≈0.13). The dashed blue horizontal line indicates the expected (target) value of the selection coefficient. *A few estimates failed at high selection coefficients because allele frequency increased so rapidly that it exceeded the 90% frequency bound before the regression window could start

**FIGURE 3:**
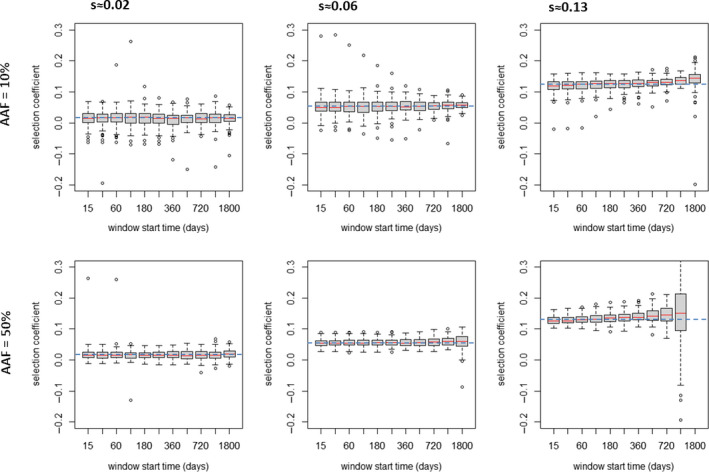
How choice of day (i.e. after introduction of the advantageous allele) used to start the regression window affects estimation of selection coefficients. The *x*‐axis shows time postintroduction that the regression window started for values of 15, 30, 60, 120, 180, 240, 360, 540, 720, 1,080, 1,800 days. The boxplots each summarize 98–100 estimates* of selection coefficients obtained by simulating 10,000 humans with malaria prevalence of 15% using a regression window with duration of 720 days. Left column: the advantageous allele encodes a drug resistance increase of 1.1‐fold. (*s*≈0.02). Central column: the advantageous allele encodes a drug resistance increase of 1.4‐fold (*s*≈0.06). Right column: the advantageous allele encodes a drug resistance increase of 2.4‐fold (*s*≈0.13). Top row is starting advantageous allele frequency (AAF) of 10%, and the lower row is starting AAF of 50%. Bottom right panel has two anomalous results: the penultimate column has larger variation because at AAF of 50% and *s*≈0.13; then, our boundary condition of AAF > 90% resistance is often exceeded during the 720‐day regression window so regression is performed over much shorter periods; the last column is empty because all runs had exceeded the AAF > 90% boundary at the time the window started so no regressions could be performed. The dashed blue horizontal line indicates the expected (target) value of the selection coefficient. *A few estimates failed at high selection coefficients because allele frequency increased so rapidly that it exceeded the 90% frequency bound before the regression window could start

**FIGURE 4:**
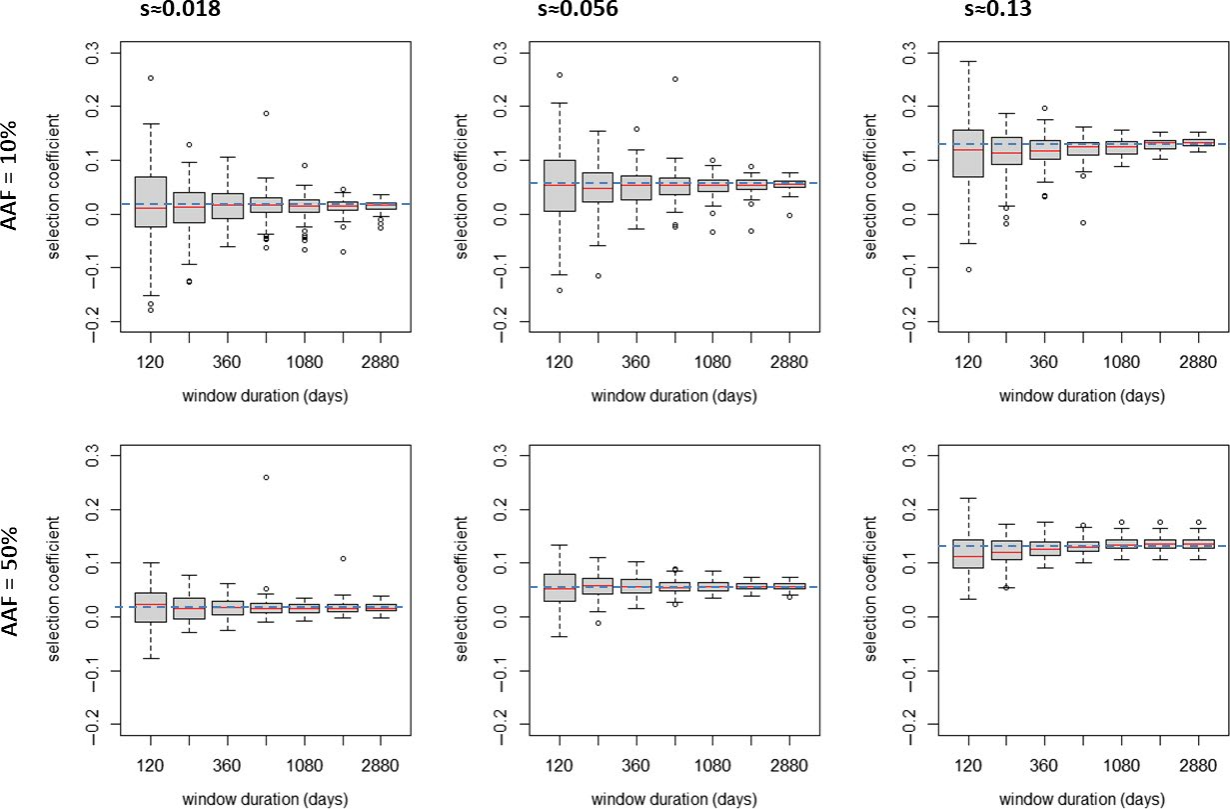
How duration of regression window affects estimation of selection coefficients. The *x*‐axis gives duration of regression window and values of 120, 240, 360, 720, 1,080, 1,880 and 2,880 days were investigated. The boxplots each summarize 98–100 estimates* of selection coefficient obtained by simulating 10,000 humans with malaria prevalence of 15% using a regression window that starts 60 days after introduction of the advantageous allele; left column: the advantageous allele encodes a drug resistance increase of 1.1‐fold (*s*≈0.018). Central column: the advantageous allele encodes a drug resistance increase of 1.4‐fold (*s*≈0.058). Right column: the advantageous allele encodes a drug resistance increase of 2.4‐fold (*s*≈0.13). Top row is starting advantageous allele frequency (AAF) of 10%, and the lower row is starting AAF of 50%. The dashed blue horizontal line indicates the expected (target) value of the selection coefficient. *A few estimates failed at high selection coefficients because allele frequency increased so rapidly that it exceeded the 90% frequency bound before the regression window could start

Each OpenMalaria simulation was run 100 times for each resistance level with different random number seeds to check consistency of estimates over “identical” runs. Selection coefficients were obtained by linear regression of ln[*p*/(1 − *p*)] as described above. We set frequency boundaries to avoid the regression tracking small numbers of alleles which would occur at high or low frequencies. The reasoning is that outside these boundaries, there may be a relatively small number of alleles of one type and stochastic variation in their number may obscure the deterministic change in their frequency, that is the effect of genetic drift as described above. The upper boundary was an advantageous allele frequency (AAF) >90% in all simulations. The lower boundary depended on initial AAF: it was AAF < 30% if initial AAF frequency was 50%, AAF < 1% if initial AAF was 10% and AAF < 0.1% if initial AAF was 5% or 1%; note that the latter two initial AAF values were only ever used to produce the data shown on Figure 2. The regression was terminated if the AAF fell outside these boundaries and regression only used data between the initial AAF frequency and the point at which AAF first fell outside the boundaries. These boundaries worked well for our simulations (>98% of simulations provided estimates of selection coefficients within these boundaries; see captions of Figures 2, 3, 4) but should be checked for the IBMs being used. We also exclude estimates of selection coefficient obtained from regressions based on fewer than five datapoints. Notably, this strategy can cause bias as runs with strong selection coefficients may be ignored as they may exceed the upper boundary or have <5 datapoints in the regression before they exceed this boundary; hence, checks should be made to ensure that a significant proportion of simulations are not being removed for these reasons.

#### 2.1.1 Decision #1: What is the appropriate starting allele frequency in the simulation?

Most advantageous alleles start at very low frequencies, and most of the timescale of spread occurs at very low frequencies before its clinical impact becomes apparent (the period of complacency; Hastings, 2001) as shown on Figures 1a. Ideally, we would investigate spread at low frequencies, but it is generally extremely difficult to track these very low frequencies in individual‐based simulations because of the effect of stochastic variation described above. A biologically reasonable starting frequency might be 10^–5^ meaning one in 100,000 infections have the advantageous allele but this is obviously impossible if the simulation, for example, has only 10,000 infected individuals. Even if the simulation did track 100,000 infected individuals, it would still be problematic to introduce an allele at a frequency of one in 100,000 as the single infection would exhibit considerable stochastic variation in its subsequent number of secondary, tertiary etc. infections for reasons described earlier, that is, for example, if *s* = 0.1 then the infection cannot leave exactly 1.1 offspring but 1.1 will be an average of a distribution with 0,1,2,3,4… secondary infections. The key computational requirement is that small numbers of resistant infections need to be avoided. Thus, a decision needs to be made on starting frequency that ensures a reasonable number of each type of allele such that stochastic variations due to genetic drift are minimized (i.e. the dynamics are largely driven by deterministic processes rather than stochastic fluctuations).

The dynamics of Figure 1 assume constant selective advantage over time. This assumption may hold at very low frequencies where small changes in frequency, say from 10^–6^ or 10^–5^, do not significantly affect malaria epidemiology. Once frequencies become larger, the epidemiology may begin to change because of the presence of the advantageous allele; for example, spread of a *hrp2*‐deletion allele might allow malaria to resurge, treatment rates to fall and prevalence to increase. We show how selection coefficients may differ depending on epidemiology in Table 1 which shows the impact of a large, but illustrative, 10‐fold reduction in malaria prevalence. The selection coefficient is almost 10 times higher when malaria prevalence is 15% than when prevalence is 1.5%. The most likely explanation is that with a higher transmission rate, people are being infected more regularly, and thus, more people are accessing treatment per unit time thus increasing the selection pressure for drug resistance (the percentage of people with detectable drug level is 35% in the 15% prevalence group falling to 8% in the 1.5% prevalence group). The proportion of people in the population with subtherapeutic drug levels is an important driver of resistance (see box 1 of Hastings & Watkins, 2006 and box 2 of Hastings, 2011 for discussions) so higher selection coefficients are generated when there are higher proportion of patients with detectable drug levels. A 10‐fold change in prevalence is obviously a very large epidemiological change but serves to illustrate our point about the potential impact of changing epidemiology on estimates of selection coefficient.

There are therefore two conflicting considerations in choice of initial AAF. Ideally, it should be as low as possible to reflect the condition under which most selection occurs (Figure 1) but this is weighed against the benefits of higher frequencies in reducing stochastic noise in allele spread. We examined different values of starting frequencies as shown in Figure 2, and values of 10% and 50% are also compared in Figures 3,4 and Figure S3. We decided on 50% as the default value to minimize stochastic change because Figure 2 shows it greatly reduces the variation in estimating selection coefficient. Also because, at least in our simulations, it did not appear to affect the epidemiological of infection (e.g. prevalence) and because our diagnostic plots (e.g. Figure S7) showed linearity of selection pressure. Note, however, that when values of *s* are sufficiently high that they reliably dominate genetic drift (*s* > ~0.1 in our default simulations based on 10,000 humans with 15% malaria prevalence) then an initial frequency of 10% can be used; this allows regression to be measured before AAF exceeds the 90% threshold applied in our analyses. We discuss the implications of this choice of initial frequency later (i.e. Recommendation #4 in Section 3) and recommend that researchers explicitly discuss how their results obtained at high frequencies are likely to also apply to selection at much lower frequencies.

#### 2.1.2 Decision #2: How long after introduction of the advantageous allele should we start measuring the slope of ln[*p*/(1 − *p*)]?

Decision #1 means we have to introduce the advantageous allele at a relatively high frequency which will inevitably change disease epidemiology, so the second decision is how long after the introduction of the advantageous allele should we start to measure “*s*”? The best policy would be to start measuring selection as soon as possible after its introduction to minimize the impact of any epidemiological changes. Plausible start times could be immediately after introduction, after 20 days (to allow secondary infections to be acquired by the mosquito, develop in the midgut, be inoculated and become patent in humans), 60 days (the estimated malaria generation, see above) and so on. We investigated this by varying the start time and quantifying its effect on estimated selection coefficients. Example results using the default duration of ln[*p*/(1 − *p*)] regression of 720 days are shown on Figure 3 but the results were fairly consistent across other durations of regression (Figure S1). Start times >60 days do not appear to bias the median estimate of “*s*” nor does it make a big difference to the coefficient of variation around the estimates. We therefore selected a delay of 60 days, equivalent to a single malaria generation, to allow transmission to stabilize.

#### 2.1.3 Decision #3: How long should we regress the slope of ln[*p*/(1 − *p*)]?

The aim is to make the duration of regression sufficiently short that the epidemiology over that time period has not changed significantly due to the introduction of the advantageous allele, while ensuring that the duration is sufficiently long that accurate estimates of “*s*” can be obtained from the regression. We investigated this effect by altering the duration of regression and quantifying how it affects the estimation of selection coefficients, see Figure 4 and lower panel of Figure S2, assuming, as justified above, that the regression starts 60 days after introduction of the advantageous allele. The duration does not appear to bias the mean or median estimate of “*s*” but did affect the variation in the estimates, the variation decreasing as duration increased (Figure S2), presumably because more datapoints enter the regression. We chose 720 days as the duration of our regression as it returns stable estimates of “*s*” with relatively small coefficients of variation (Figure 4). We confirm estimates of selection coefficient and IQR are robust over these combinations of staring time and duration of measurement in Figures S4 and S5.

Finally, we needed to confirm that spread of the advantageous allele had no significant impact on epidemiology (or, if it did, that it did not alter the magnitude of “*s*”) so that the value of “*s*” from our regression will be a valid approximation for that occurring during the critical period of spread from low initial frequencies. Figure S1 shows that the estimated selection coefficient did not systematically change with time since introduction of the advantageous allele; frequency will have increased over this time implying that estimates of selection coefficient were not affected by allele frequency.

#### 2.1.4 The impact of these decisions

There appear to be no objectively “correct” solutions to these three decisions, all of which incur trade‐offs. It may be possible to tailor the decisions to specific circumstances. For example, if selection coefficients are known to be large, and it is computationally feasible for the simulation to track a large number of infections in a reasonable time frame, then the advantageous allele may be introduced at a much lower starting frequency to minimize its epidemiological impact after its introduction. We wished to avoid using different methods in different circumstances and wanted to identify a robust method which we believe will be applicable over the wide ranges of parameter spaces explored by OpenMalaria; hence, we settled on the above methodology. We are not prescriptive on the methodologies to be employed but herein describe what trade‐offs we encountered and how we addressed them; importantly, these will almost inevitably occur when tracking genetic selection in IBMs of most infectious diseases. We used OpenMalaria as a case study in decision‐making which people using other IBM for other alleles, may find useful.

In summary, the default method we use for estimating “*s*” in OpenMalaria is as follows. The advantageous allele is introduced at 50% frequency (with the caveat that if “*s*” is very high, starting frequency of 10% gives a longer duration of regression before frequency exceeds our limits of 90% which may improve accuracy). The regression then starts 60 days after the introduction of the advantageous allele (this one‐generation time lag allows malaria transmission stages, gametocytes, to mature and reflect the preferential transmission of the advantageous allele) and continues over 720 days (2 years) which assuming six generations per year (see below) is 12 malaria generations. OpenMalaria outputs data every 5 days, so the full regression period of 720 days provides 144 datapoints for the regression. We then run repeated stochastic realizations to estimate a distribution of our estimates (Figures 2, 3, 4) or calculate means and coefficients of variation (Figures S1–S3) to confirm that we have reasonably stable estimates of “*s*.”

### 2.2 Incorporating mutations into the IBM

We argued that selection coefficients be measured when advantageous alleles are present at high frequency (to minimize the impact of genetic drift) and used to extrapolate spread of the advantageous allele over the whole period of selection. This period of selection can start from any frequency, so a key question is what starting frequency is most appropriate for this extrapolation. In some circumstances, we may be able to simply assume that advantageous alleles are already present at a given initial frequency, for example at a mutation/selection equilibrium. In these circumstances, it is straightforward to use the estimate of “*s*” to track their subsequent spread from their initial, presumably very low, frequency. For example, if this initial low AAF is *p*(0), then the frequency after “*t*” generations, *p*(*t*), can be obtained by substitution as in the following equations. Figure 1b shows that

(1A) $$\ln\left[\frac{p(t)}{1‐p(t)}\right]=\ln\left[\frac{p(0)}{1‐p(0)}\right]+st.$$

Or, equivalently,

(1B) $$\frac{p(t)}{1‐p(t)}=\frac{p(0)}{1‐p(0)}e^{\mathrm{st}}.$$

Noting that if the odds of *p*(*t*) are “*x*,” that is *p*(*t*)/[(1 − *p*(*t*)] = *x*, then the allele frequency at “*t*” is given by *p*(*t*) = *x*/(1 + *x*)].

Or, alternatively, the time taken to reach a frequency of *p*(t) from *p*(0) can be obtained by substitution as

(2) $$t=\frac{\ln\left[\frac{p(t)}{1‐p(t)}\right]‐\ln\left[\frac{p(0)}{1‐p(0)}\right]}{s}.$$

Similarly, the selection coefficient can be obtained from field data reporting frequencies at different times (a common form of data; see Recommendation #1 of Section 3) as

(3) $$s=\frac{\ln\left[\frac{p(t)}{1‐p(t)}\right]‐\ln\left[\frac{p(0)}{1‐p(0)}\right]}{t}.$$

Alternately, the assumption may be that the advantageous alleles are not yet present in the malaria population, in which case their de novo input by mutation must be incorporated into the IBM. The problem then is to track the origin and spread of such mutations while avoiding having very low numbers of the advantageous allele within the IBM. We suggest the following strategy to allow the input of de novo mutations into the IBM as a four‐stage process.

1. Define “mutation rate,” *μ*, as a user‐defined input into the simulation. We define this mutation rate per inoculum, *μ*, which in our malaria IBM is the probability that a single mosquito bite delivers an inoculum consisting solely of parasites containing the advantageous allele. This allows details of how mutations arise to be studied external to the IBM (see Recommendation #3 of Section 3). Importantly, quantifying the input of new mutations as *μ* simplifies the introduction of mutations into IBMs and reduces the stochastic element of their input and improves comparability between replicate runs.
2. Use the selection coefficient obtained above to calculate the probability that the mutation successfully survives chance extinction in the first few transmissions after its introduction. This is discussed in more detail elsewhere (Hastings, 2004) but the basic result is that if reproductive success follows a Poisson distribution, then the probability that the de novo mutation survives to become established in the population is approximately 2*s* when *s* < ~0.1 (Charlesworth & Charlesworth, 2010; Crow & Kimura, 1970; Haldane, 1927). Reproductive success is generally not Poisson and is much more overdispersed; if we quantify this overdispersal by a negative binominal distribution, as is commonly used in infection transmission, then probability of establishment may be reduced substantially (table 1 of Hastings, 2004 and see Parsons, Lambert, Day, and Gandon, 2018 for a more sophisticated discussion). We denote the probability of a novel mutation surviving chance extinction as Ψ and can estimate it as

(4) Ψ=s,

based on table 1 of Hastings (2004) which shows this relationship holds for values of *s* from ~0.01 to ~0.1 and a negative binomial distribution of transmissions (a characteristic of most parasite infections) with a dispersal parameter of 0.1 (moderately overdispersed). Alternative estimates of Ψ can be obtained by simulation under different assumptions of dispersal parameter (e.g., *s*/5 may be a better estimate of survival probability when transmission is highly overdispersed, e.g. k = 0.1 in table 1 of Hastings (2004), but this simple relationship serves its illustrative role here.

1. We can then calculate the expected number of new mutations entering the human population per (malaria) generation, θ, as

(5) θ=μΨλ,

where *λ* is the number of successful transmissions in the IBM per mosquito generation. This depends on the FOI which is conventionally quantified as the number of successful (i.e. resulting in a new, viable infection) inoculations per human per time unit. If we set the time unit as being a malaria generation, then *λ* = FOI*N where *N* is the number of humans in the simulation. Note that FOI is typically much lower than the infective contact rate because many contacts do not produce a viable infection due to factors such as low inoculum size and acquired immunity in the human. In vector‐borne disease, the contact rate is usually quantified as the EIR, that is the average number of infective bites per human per time period. In the specific case of malaria, Smith, Killeen, et al. (2006), and Smith, Maire, et al. (2006) established a relationship between EIR and FOI which, since malaria EIR is commonly estimated in the field, enables IBMs of malaria to be calibrated against field data when attempting to incorporate mutational input; we used the method described in that paper.

**FIGURE 5:**
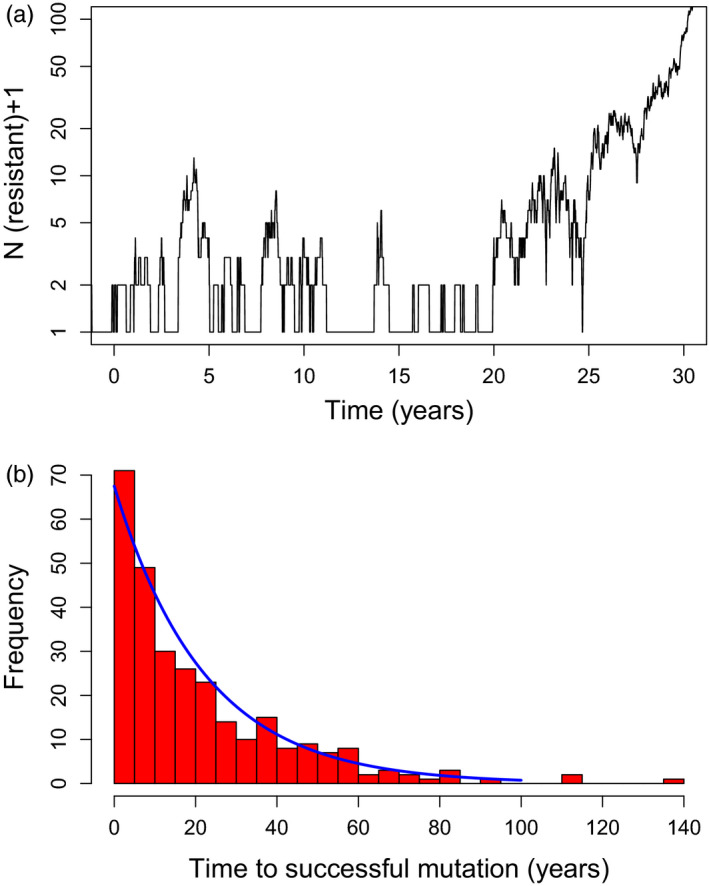
Validation of the theoretical approach for incorporating spontaneous mutations using OpenMalaria simulations; see Supporting Information for calibration. (a) An example from OpenMalaria simulations showing the random arrival and stochastic loss of resistance mutations. Note that the *Y*‐axis is the number of resistance infections *plus one*. Many time points have zero infections but log(0) is mathematically undefined so a common strategy in these circumstances is to add 1 to all values. The baseline of zero resistant infections is therefore plotted as “1” in the figure. Most mutations only generate a single resistant infection (value of 2 on the scale) before being lost, notably the 7 mutations that occurred between 16 and 19 years. This rapid loss is not inevitable and the mutation that occurred around 3.5 years generated 12 infections at its peak before being lost, and the one at 20 years generated 18 at its maximum. The mutation at year 24.9 did survive stochastic loss and became established in the population. (b) Histogram showing observed times until establishment of a de novo mutations obtained from 280 runs of OpenMalaria calibrated as described in this Supporting Information. The solid blue line is the expected fit from Equation (6)

Equation (4) enables us to calculate the distribution of “wait times” until a mutation successfully enters the population; this is an exponential decay, rather than a normal distribution, see Figure 5b, that is

(6) $$f\left(t\right)=\theta e^{‐\theta t}.$$

Sampling from this distribution provides the time (in generations) until a mutation first successfully enters the malaria population tracked in the IBM.

1. Having obtained a wait time, we can then use “*s*” to calculate its subsequent spread until it reaches any given point, for example time to reach 5% frequency. Assume, for example, that the IBM is tracking a population of 1,500 infections (note it is number of infections, not number of humans), then the initial frequency *p*(0) of a de novo mutant is 1/1,500. The distribution of times for a de novo mutation to successfully enter the population and subsequently reach any given frequency p(t) can be simply obtained by summing the wait time for its successful input into the population (sampled from Equation 6) and the time for its subsequent spread to *p*(*t*) (Equation 2). The implicit assumption in this calculation is that the rate of de novo mutations entering the population is small compared to the selective advantage (i.e. *θ* ≪ *s*), so that the spread of the first mutation dominates any later input of new mutations, meaning that we need only track the spread of the first mutation. If this is not the case, the calculation for spread can still be made but allowing recurrent, sporadic de novo mutations to enter the population using Equation (5).

We used OpenMalaria to validate the theoretical approach described above (details in Supporting Information). Figure S6 demonstrates the difficulty of incorporating the dynamics of a mutation with a de novo rate of 10^–7^ per infection per generation. An uncritical approach using small populations sizes to investigate very large populations would result in extremely large underestimates of mutational input: the wait times are much longer in small populations, and this dominates their subsequent rate of spread within smaller population. Figure 5 shows results obtained from the IBM. Figure 5a shows that, as expected, a large proportion of advantageous de novo mutations are lost by stochastic fluctuations within a few generations of their arrival. Figure 5b shows how the wait times until successful establishment of a de novo mutation matches the distribution predicted in Equation (6).

There are two clear advantages of this four‐stage strategy of incorporating mutations. Firstly, it allows biological details of how mutations occur to be removed from within the IBM which allows better comparability between studies (see Section 3, recommendation #3). Secondly, it removes the temporal variation in wait times for mutational input from the IBM and places it in Equation 5. It is obviously easier and much faster to repeatedly sample from the distribution in Equation 5 than to repeatedly run replicates of the IBM to capture and quantify this stochastic variation in mutational input.

### 2.3 Identify the power of the IBM to accurately estimate small selection coefficients

In Part 2.1, we deliberately focussed on selection of sufficient strength that selection was able to dominate genetic drift in our simulations, that is selective coefficients > 2% and a malaria population census size, *N*, of ~1,500 (i.e. 10,000 humans tracked with a malaria prevalence of 15%).

IBMs typically simulate humans. If disease prevalence is low in near‐elimination scenarios, then, by definition, the pathogen population size may be small. Suppose a simulation of 10,000 humans with a pathogen prevalence of 1% gives a pathogen population size of 100. This may seem large but, as described earlier, it is effective population size, *Ne*, that is important rather than the observed or “census” population size, *N*. The reason this is so important is that *Ne* sets the sensitivity of the IBMs to detect selection acting on advantageous alleles. As *Ne* becomes small, random frequency changes due to genetic drift become so large that they can obscure selection processes. There are various approximations for deciding whether selection or drift will be the dominant dynamics driving changes in allele frequency but, generally, drift dominates when *s* ≪1/*Ne* and selection dominates when *s* ≫1/*Ne*, and both play important roles when *s* and 1/*Ne* are around the same magnitude. Suppose selection coefficient is 0.02 which is moderately strong, that is a 2% increase per generation, this suggests it will only dominate drift when *Ne* ≫ 50.

**FIGURE 6:**
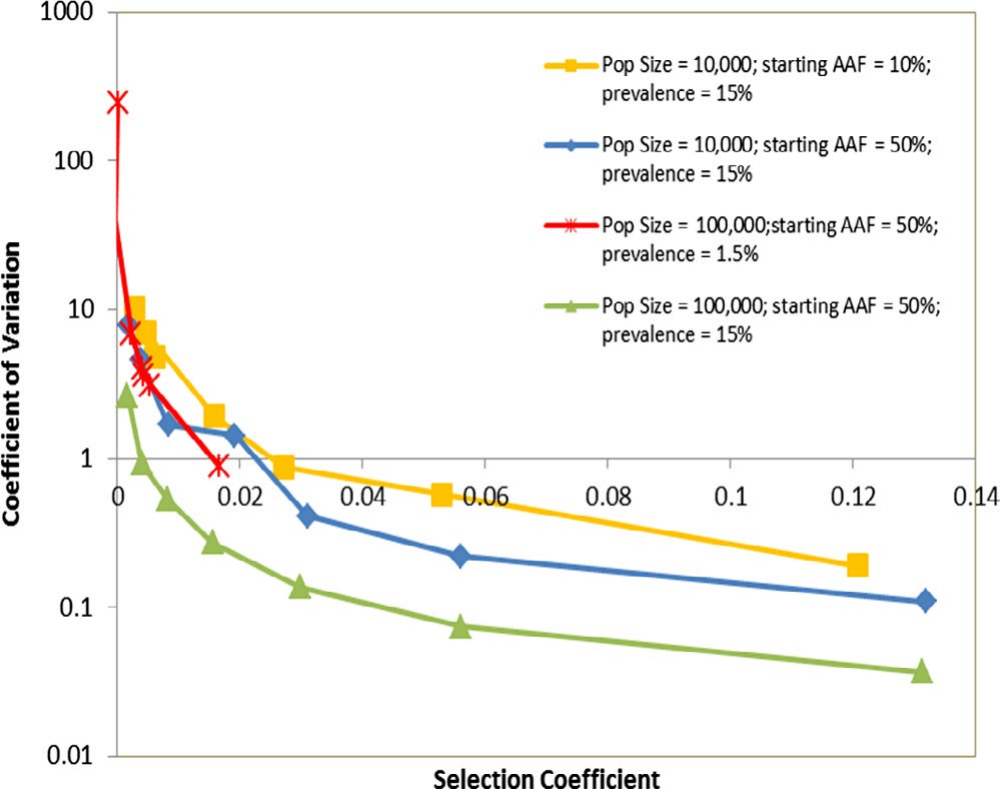
How the power of a simulation to detect small selection coefficients depends on its size. Four parameterizations were investigated: (a) The default simulation size, that is 10,000 humans with a malaria prevalence of 15%; starting advantageous allele frequency (AAF) of 10%; (b) The default simulation size used in this work, that is 10,000 humans with a malaria prevalence of 15%; starting AAF of 50%; (c) Simulation of 100,000 humans with a starting AAF of 50% and malaria prevalence of 1.5%, meaning malaria population size is nearly identical (there will be stochastic differences) to the second parameterization where population size is 10,000 but prevalence is 15%. (d) Simulation of 100,000 humans with a starting AAF of 50% and malaria prevalence of 15%, that is a 10‐fold increase in malaria population size compared to the default size of 10,000. Each parameterization was run 100 times and selection coefficient estimated by regressing over 720 days starting 60 days after introduction of the advantageous allele. Note that the third parameterization (red line) is associated with lower selection coefficients (see Table 1) and consequently is only plotted at the extreme left of the *x*‐axis

The problem is that, except in very simple cases, there are no algebraic means of converting the “census” population size observed in IBMs, into the equivalent *Ne*. One option would be to track neutral (i.e. nonselected) genetic markers in the simulation and use the level of linkage disequilibrium to estimate *Ne* (e.g. Waples & Do, 2010). However, it is probably more informative to empirically estimate the limit of sensitivity of the IBM to detect small selection coefficients. We demonstrate this by further reducing the resistance level (IC50) of the advantageous alleles below that investigated in Part 2.1, that is fold increases in IC50 are less than 1.1 (Table 1). The dynamics of spread when selection coefficients are low is shown on Figure 6 and shows the coefficient of variation around mean estimates of “*s*” (cf Figures 3 and 4 where selection is higher and dominates drift). Figure 6 has the following pattern:

The top line was obtained from the following simulation:

- 10,000 patients with malaria prevalence of 15% and starting mutant frequency of 10%; *N* = 1,500

There are then two lines that are effectively superimposed, that is

- Increasing the starting AAF to 50%; this increases sensitivity compared to the top line, presumably by reducing stochastic variation in allele number.
- Increasing population size to 100,000 and decreasing malaria prevalence to 1.5%; this gives the same malaria census population size as 10,000 people with 15% prevalence (i.e. *N* = 1,500) so it is an excellent demonstration that it is the malaria population size being tracked *not the human population size*, that is the critical factor governing ability of the IBM to estimate selection coefficients.

Finally, there is a lower line obtained from the following simulation as follows.

- 100,000 patients with malaria prevalence of 15% and starting mutant frequency of 50%. This has *N* = 15,000, that is a 10‐fold greater malaria population size compared to the 100,000 population with 1.5% prevalence. As expected, the ability of the IBM to accurately estimate selection coefficients is greatly increased.

As expected, the accuracy of estimation does increase as the number of infections tracked is increased, either by increasing the number of patients tracked and/or by increasing malaria prevalence (Figure 6). In the specific case of default simulations of 10,000 humans with 15% malaria prevalence (blue line of Figure 6), they are unable to accurately estimate values of “*s*” less than about 0.02 because the CV increases to the extent that a large number of replicates would be required to get an accurate standard error around the estimated value of *s*. Increasing population size to 100,000 enables estimates of *s* down to around 0.005 (green line of Figure 6). We base this limit on a CV < ~1 but this is arbitrary and illustrative and, in reality, the limit depends on how many replicates can be feasibly run. A larger number of runs will reduce standard error around the estimates but, importantly, it may be more computationally efficient to run fewer, large simulations, rather than a larger number of small simulations. Recall that standard error around the mean (*SEM*) is given by.

$$\text{SEM}=\frac{\sigma }{\sqrt{n}},$$

where σ is the standard deviation of estimates and n is sample size. If we take the central point plotted in Figure 6 (i.e. *s* = 0.0156; see Table 1) with 50% starting frequency and 15% prevalence, then a population size of 10,000 has a CV of 1.43 (Figure 6) resulting in *σ* = 0.022 and a population size of 100,000 has a CV of 0.27 (Figure 6) resulting in *σ* = 0.0042. Assuming the computation time scales with the chosen population size (i.e. a run tracking 100,000 individual requires 10 times more run‐time than a population of 10,000), then a smaller number of simulations tracking a larger population provides better accuracy. For example, 100 simulations of 10,000 individual give *SEM* of 0.0022, while 10 runs of 100,000 give a *SEM* of 0.0013. Similarly, 50 runs of 10,000 give a *SEM* of 0.0032, while five runs of 1,000,000 give a *SEM* of 0.0018. It is therefore likely that running exploratory trial runs to establish the sensitivity of the IBM to detect values of *s* around the anticipated value could lead to optimized IBM simulation strategies that can significantly reduce the standard errors associated with parameter estimates.

The main factor that reduces *Ne* below the census size is the differences in reproductive success of different infections (in malaria, most plausibly due to some people being bitten more frequently than others, and acquired human immunity affecting an individual's infectivity and susceptibility). Figure 6 therefore demonstrates the general principle although the exact quantitative relationship between census population size and *Ne* will depend on the IBM and its calibration: adding factors that further increase this heterogeneity in reproductive success will further reduce *Ne* and hence the ability to detect low magnitudes of *s*. Consequently, the ability of IBMs to detect low values of *s* is not simply dependent on population size, but on their structure and calibration; this emphasizes the need to continually check sensitivity of IBMs before drawing conclusion of magnitude of selection coefficient.

## 3 DISCUSSION

Individual‐based models for complex disease such as malaria have an intrinsic trade‐off: the more realistic and complex the biological and clinical description of transmission and disease, the more computational power is required to simulate populations, and the smaller the number of patients/infections that can be tracked in a reasonable timeframe. This is not usually an issue for simulating the overall epidemiology (although stochastic loss of the pathogen population may occur at low prevalence), and IBMs of malaria have been successfully used to investigate a number of control measures as described in the Introduction. However, the finite sizes of IBM do affect their ability to incorporate genetic selection of advantageous alleles. In the specific case of malaria populations, such alleles evolve to counter control interventions such as those encoding drug resistance, vaccine insensitivity and RDT escape mutations. Given the likely impact of these alleles, a strategy to incorporate such genetic processes into IBMs is urgently required, and to our knowledge, this is the first attempt to explicitly do so. We make four specific recommendations that may help other researchers attempting to do this.

### 3.1 Recommendation #1. Report selection coefficients and validate the values obtained from IBM against field data

We strongly recommend that IBMs be interrogated to report the selection coefficient, *s*, of the advantageous allele. This is a common scale which can be used to compare results from different IBMs and which can be used to calibrate/validate against field data which typically report selection coefficients acting on alleles (see drug resistance examples below). It is the central population genetic parameter quantifying the response to selection (e.g. Charlesworth & Charlesworth, 2010; Hartl & Clark, 2007) and is widely extracted and reported from clinical and/or field data. For example, selection for/against antibiotic resistance is widely measured on this scale, see for example: Levin et al. (1997); Greenfield et al. (2018); box 3 of Melnyk et al., (2015); figures 2 and 3 of Gullberg et al. (2011)). Selection coefficients also affect other genetic process as was briefly described elsewhere (Nsanzabana et al., 2010), that is they determine rate of geographical migration rate, survival probability of new mutations (as described above), the genetic impact of selective sweeps and determines the frequency of resistance prior to the introduction of a novel drug as a mutation/selection balance.

As an example of IBM validation using selection coefficients, we searched the literature for estimates of selection coefficients associated with alleles encoding malaria drug resistance in malaria and found these range from around 0.02 to 0.12 as in the following examples (which we do not claim to be exhaustive). Anderson and Roper (2005) reported selection coefficients of 0.05 and 0.076 for two *dhfr* alleles and ∼0.13 for a *dhps* allele. Anderson (2004) collated data suggesting values of *s* ranging from 0.03 to 0.11 for the same mutations. Estimates from SE Asia are *s*~=0.11 for *dhfr* (Nair et al., 2003) and *s*~0.08 for *kelch*13 (Anderson et al., 2016). Nwakanma et al. (2014) reported values of *s* as 0.15, 0.13, 0.11 and 0.11 for *crt*, *mdr*, *dhfr* and *dhps,* respectively, over a 25‐year period in Gambia; note that they assumed 2 generations per year so, for consistency with the assumption of 6 generations per year, these values should be divided by three giving estimated in the range 0.03–0.05. Nsanzabana et al. (2010) reported values of *s* = 0.02–0.6 for 3 loci undergoing selection over a 12‐year period in Papua New Guinea. Finally, Okell et al. (2018) investigated a range of positively and negatively selected resistance alleles and found positive selection coefficients ranging up to a median of 0.023 for the most selected allele (*mdr*1‐184F under selection from the drug artemether–lumefantrine; note they assumed three malaria generations per year so should be divided by two to convert to our scale of six generations per year). The magnitude of selection coefficients depends on the type and amount of drug deployed and, arguably, the number of malaria generations per year (which depends on local transmission patterns) but, generally, IBMs producing values in this range are re‐assuring, while those producing higher or lower values may require supporting clarification about why they produce results different from field observations.

Some authors report basic genetic outputs from the models, for example Watson et al. (2017) report the spread of *hrp*2 deletions from various starting frequencies in their Figure 1, while some authors do not report the dynamics of mutational spread and simply report epidemiological/clinical impact (e.g. Nguyen et al., 2015). Simple description of genetic spread and/or clinical impact is informative but we argue strongly that the dynamics should also be described by a selection coefficient as this allows a reader to easily predict spread from his/her own choice of initial frequency and is a common genetic scale on which to compare selection processes. Selection coefficient can be extracted from previous work using our approach. For example, Watson et al. (2017) show the increase in frequency of *hrp*2 deletion over time in their Figure 1. The change in frequency between values of 0.25–0.75 appears linear for most of their plots (in line with our results, Figure 1a of this paper) and generally takes 5 years, equivalent to 30 malaria generations, to spread from 0.25 to 0.75. Substituting these values into our Equation (3; i.e. *p*(0) = 0.25, *p*(*t*) = 0.75, *t* = 30) gives a value of *s* = 0.07 which is highly consistent with “*s*” observed for drug resistance, presumably because the underlying selective forces are similar, that is drug resistance mutations survives treatment, while *hrp2* deletions simply avoid treatment. This example clearly shows how reporting “*s*” can effectively summarize a whole graph of data (i.e. figure 1 of Watson et al., 2017) while also allowing easy comparisons between studies.

We have reported selection coefficient in units per generation, even though OpenMalaria runs in five‐day timesteps with overlapping generations. The per generation timescale is a population genetics convention but can be easily interconverted to units per day, or perhaps more convenient, per year (it only has to be in units per generation in this work for application of Equation 4). An annual timescale is robust (and hence should be reported) as it removes assumptions about durations of generations that may differ between studies (e.g., Anderson, 2004 assumed six generations per year but Nwakanma et al. (2014) assumed two generations per year). There may also be subdecisions of timescale, for example selection coefficients acting on antibiotics may be reported on a scale of cell cycles, “generations” (which we take to mean transmissions between hosts), or annual basis. An annual rate may also be more appropriate if seasonal variation in transmission causes fluctuations in selection coefficient over the year. The annual rate of spread is, of course, the one most useful to policy makers aiming to mitigate its spread. We do, however, recommend that selection coefficients be reported even if their estimation is not the primary objective of the study For example, Nguyen et al. (2015) reported how selection of malaria drug resistance affected clinical outcomes such as treatment failure rates. The clinical predictions are important (e.g., WHO mandate the replacement of first line antimalarial drugs if failure rates exceed 10%) but we believe if they did report underlying selection coefficients, it would support comparability between studies and also enable validation that their predicted dynamics are consistent with field estimates describing selection for resistance.

### 3.2 Recommendation #2. Use the magnitude of “*s*” to optimize the computational approach

Any IBM of finite size will inevitably have a lower limit below which “*s*” cannot be estimated with precision due to the presence of genetic drift; see Figure 6. A first step would therefore be to empirically estimate its power and avoid trying to obtain estimates of “*s*” below this lower limit. This is particularly relevant where extensive explorations of parameter space are planned, as it may be inevitable that small values of “*s*” may be encountered and need to be avoided, or possibly discounted in subsequent analysis as being unreliable. In these circumstances, it is also beneficial to optimize the computational approach as detailed earlier, that is decide whether it is more computationally efficient to run a smaller number of replicates of larger IBMs or vice versa.

### 3.3 Recommendation #3. Consider how best to incorporate mutations

If mutations are already present in the population before deployment, presumably in mutation/selection balance (e.g. for malaria see equation 2 of Hastings, 1997), they can be simply incorporated as starting frequency *p*(0) in Equations (2) and (3). However, if new de novo mutations are to be incorporated into the IBM, it is best to try and make details of how mutations arise external to the simulation and summarize their input into the IBM as simple mutation rate per inoculum. In particular, use of Equation (5) to incorporate wait times for de novo mutation to enter the population is far more efficient than using repeat runs of the entire IBM to achieve the same purpose.

How mutations give rise to advantageous alleles may be relatively noncontentious in some situations. For example, deletions in *hrp*2 or the mutations that result in altered antigenic profiles in vaccine‐escape alleles presumably reflect relative well‐characterized eukaryote deletion rates and codon mutation rate, respectively. In the example of drug resistance mutations in malaria, this is extremely unclear. Hastings (2004) identified four possible sources of mutations that may be selected by drug deployment: (a) mutations already present as a mutation/selection balance prior to drug deployment a; (b) mutations selected de novo from among malaria infection undergoing drug treatment; (c) mutations selected de novo from new infections emerging from the liver and encountering drugs persisting from previous treatments; and (d) spontaneous mutations that occur in untreated humans or in the mosquito stages that are subsequently inoculated as resistant. The relative importance of each is unknown and may even vary between drugs (e.g. resistance to atovaquone can be observed being selected from among malaria infections at time of treatment in around 30% of treatments (Looareesuwan et al., 1996). Authors may differ in their underlying assumptions about how mutations arise so this strategy removes such differences from the simulations. For example, if most mutations are assumed to arise from drug‐treated infections (source (b)), then mutation rate, *μ*, would be a function of drug treatment rates and probability of mutation emergence in treated infections; if most mutations are assumed to be spontaneous mutations (source (d)), the mutation rate would simply be the spontaneous rate in mosquitoes and untreated humans, and so on. The important point is that calculation of *μ* is done external to the simulations, then simply brought into the IBM through inclusion in Equation (5). We therefore argue that the most transparent approach is to externalize assumptions about how resistance mutations arise from the IBMs and use the latter simply to track their subsequent rate of spread and likely clinical impact.

### 3.4 Recommendation #4. Explicitly discuss how selection coefficients measured in the IBM at a given starting frequency are relevant to spread at low frequencies

There is dilemma at the heart of bringing selection into IBMs: the desire to have low advantageous allele frequencies to reflect the epidemiological setting at which most selection occurs (Figure 1) and the desire to have large numbers of each type of allele to accurately estimate selection coefficients (Figure 2).

The most significant compromise we had to make in our simulations was to assume a high frequency (10% or 50%) of the advantageous allele when introduced into the IBM to ensure a sufficiently large number of each allelic type, that is advantageous and wild type. Table 1 shows that “*s*” may vary depending on the underlying epidemiology so the first check we made was to examine epidemiology outputs from OpenMalaria to confirm that epidemiology was not changing rapidly as the advantageous allele (resistance in our case) spreads. The second check was to consider if selection coefficients may rely on allele frequency. For example, the spread of drug resistance may affect intrahost dynamics complicate and even stabilize the dynamics of spread (previously discussed in Hastings (2006). The dynamics of *hrp*2 deletions provide an excellent example of how “*s*” may depend on frequency. Superinfection (i.e. simultaneous infection with two or more malaria clones) is common in areas of moderate‐to‐high transmission. *Hrp*2‐deletion mutants co‐infecting humans with wild type will be common at low frequencies and will presumably have reduced selective advantage because the co‐infecting wild type, *hrp*2‐expressing parasite may present a signal sufficiently strong for diagnosis to occur. However, as deletion frequency increases, so will the proportion of superinfections consisting solely of *hrp*2 deletions and this increases their selective advantage as they will escape diagnosis. In this case, we would argue that estimates of selection coefficients obtained at higher frequencies *hrp2* deletion are only valid if superinfection does not occur, that is there is only a single malaria clone in each host. The same phenomenon occurs if the degree of superinfection changes as a result of seasonal fluctuations in malaria transmission intensity (e.g. figure 3 of Watson et al., 2019). A suitable secondary check might be to assume a variation in MOI and work out the probability that a *hrp*2 deletion is detected and then incorporate this probability of detection into the IBM as a second check. Finally, Figure 1b suggests a useful diagnostic: if a plot of ln[*p*/(1 − *p*)] obtained over the time course of the simulation is nonlinear, it is suggestive of frequency‐dependent selective effects. Figure S7 shows typical diagnostic plots obtained from OpenMalaria; in this case, there is no evidence of nonlinearity occurring in our simulations.

## 4 CONCLUSIONS

We emphasize that the purpose of this study is to demonstrate how selection can be optimally detected and quantified in IBMs of infectious disease. In particular, we wished to avoid a distracting discussion of the nuances of exactly what factors drive malaria drug resistance and how they combine to determine the selection coefficient. However, one factor characteristic of malaria transmission in many geographical locations is seasonal variation in its intensity, usually as a result of mosquito numbers and/or longevity increasing during a rainy season, possibly accompanied by a change in vector species composition (we also note that the same effect may occur for bacteria in temperate regions where winter crowding increases transmission). The primary impact of variation in intensity of transmission will be temporal fluctuations in degree of malaria superinfection. This will alter selection pressure on some mutations, for example those encoding hrp‐2 deletions (Watson et al., 2019). It will also change the level of recombination in the malaria population which would have an impact on simulations tracking two or more loci (but would have no effect of the single‐locus dynamics tracked here). Seasonality may also have an indirect effect as drug use may increase during higher transmission periods increasing selection for resistance. If seasonality is found to have an impact, it may then be better to report selection coefficients separately for low‐ and high‐transmission seasons or on an annual basis. Finally, note that seasonality reduces the effective population size making selection harder to detect against stochastic fluctuations in allele frequency. When tracking the fate of de novo mutations, it is straightforward to use the same methodology based on selection coefficients (see discussion around Equation 4) simply noting that the expected number of resistant transmissions sampled from the negative binomial distribution in the current generation will change depending on the selection coefficient operating at that time in the annual transmission cycle (Hastings, 2004).

It is fortunate that malaria, and most bacterial and viral pathogens, are haploid, that is contain only a single copy of each gene. If diploids are considered (e.g. the diploid worms responsible for human diseases such as elephantiasis, river blindness etc) or insecticide resistance in the vectors of diseases such as malaria, sleeping sickness and tick‐borne relapsing fever, the genetics becomes much more complicated. The dominance relationship between the wild type and advantageous allele needs to be considered, and this means that the spread of advantageous alleles will not be linear on a logit scale (i.e. Figure 1b).

The results presented here were obtained using one specific IBM, OpenMalaria, but the underlying principles are universal not just to malaria, but to bringing genetic selection into IBMs of other infections (see, e.g. Bershteyn et al.(2018) for a description of a more general, open‐source IBM applicable to several important human diseases). The most implementation‐dependent recommendation is likely to be the specifics of how to measure selection coefficient, that is choice of starting frequency of the advantageous allele, how long after introduction of the advantageous allele to start regression and the optimal duration of the regression. This may vary both with the IBM's underlying structure and assumptions and also with its calibrations, for example how rapidly transmission and epidemiology stabilize after introduction of the advantageous allele. We suggest researchers using IBMs to track genetic spread follow a similar suite of analysis to ourselves. Our recommendation that measurement be delayed for one malaria generation (60 days) and to continue over the subsequent 2 years (i.e. for 20–24 malaria generations) appears robust in our simulations and seems intuitively sensible. The second approach, that of incorporating mutation externally to the simulations, should be widely transferrable across IBMs. The final methodology, to validate the IBM's ability to accurately estimate the small selection coefficient by examining CV over replicates, is also likely to be universal across platforms; it will be highly informative to see how different platforms perform in this respect. What is clear is that it is highly advisable to carry out these types of checks when incorporating genetic selection into IBMs and to report them in the subsequent publications. In particular, the recommendations we list above will apply to all IBM simulations that incorporate genetic selection, irrespective of the disease. We do not imply that the same quantitative decisions will apply to all systems (e.g. our decision to track selection for 720 days starting 60 days after introduction of the advantageous allele) but we stress that such decisions will occur in simulation outputs of other IBM either implicitly or explicitly and that they be addressed and discussed. We have tried to provide an illustrative roadmap for making such decisions and look forward to future work in the topic.

## Supporting information

Supplementary Materials

## Data Availability

The data that support the findings of this study are available from the corresponding author upon reasonable request.
