# Supplementary material for: Incorporating genetic selection into individual‐based models of malaria and other infectious diseases
Source: Evol Appl. 2020 Aug 11;13(10):2723–39. doi: 10.1111/eva.13077 (PMC7691459; doi:10.1111/eva.13077)
Supplement: Supplementary file 1 — Supplementary Materials [file EVA-13-2723-s001.docx]

**This** **supplementary information has two parts**

**SI.1 Technical details of running OpenMalaria used to obtain the results described in the main text.**

**SI.2 Incorporating de novo mutations into OpenMalaria**

**SI.3. Additional plots.**

**SI.1 Details of OpenMalaria simulations used to obtain the results described in the main text.**

We use OpenMalaria as an exemplar individual based model (IBM) that has been widely used to investigate malaria transmission dynamics and interventions (e.g. Smith et al., 2006, 2008, 2012; a full list of publications can be found at <https://github.com/SwissTPH/openmalaria/wiki/References>). OpenMalaria is a stochastic, individual-based, simulation model of malaria in humans linked to models of malaria in mosquitoes; it is opensource with extensive online documentation as described on its website. The model includes sub-models of infection of humans, blood-stage parasite densities, infectiousness to mosquitoes, incidence of morbidity including severe and hospitalisation, and mortality directly due to malaria or as a co-morbidity. A recent model variant of OpenMalaria includes explicit mechanistic model of parasite densities in the human host (adapted from the model of Molineux et al (2001)), the presence of genetically-distinct malaria clones in the same host (“superinfection”) as well as parasite genotype tracking and explicit PK/PD drug modelling.

In this work, we constrained our investigations to a relatively small number of scenarios and kept calibration of OpenMalaria as simple as possible so that the methodology can be duplicated in other IBM simulation platforms if required.

Version 40.1 of OpenMalaria does not allow the input of de novo mutations (but see part 2 of this supplementary information), so both the advantageous and wildtype alleles must be present from the start of the simulation. We therefore set an initial frequency of the advantageous allele at the start of the burn-in. In general, one would hope this input allele frequency is not altered too much by chance fluctuations during simulation burn-ins. We refer to this initial frequency as “target frequency and use boundary frequencies (described in their main text) to exclude any runs that have drifted too far from their target frequencies.

OpenMalaria version 40.1 was used to simulate all runs. The geographic scenario was based on Southern Zambia with respect to it the population demography and vector species composition. In summary, simulations were run as follows.

- A 99 year warm-up period to ensure that the level of transmission, immunity, vector bionomic parameters, human age distributions, and so on are all at approximate periodic steady states before the introduction of interventions.
- An initial 10 year “burn-in” period. The 99 year warm up does not output any data (to avoid slowing the computation and to save space) so the 10 years monitoring of the population was used to start outputting data and to check epidemiological stability had been reached. Dihydroartemisinic+ Piperaquine (DHA+PPQ) was the only drug deployed for routine use in the health care system and parasites were fully sensitive to DHA+PPQ. Access to treatment was set to 40% and there was no self-treatment by any antimalarial drug outside of official treatment sources. Patients seeking treatment were tested for malaria infections by microscopy (diagnosis in OpenMalaria is probabilistic with a 50% chance of diagnosis when parasite density reached 20 parasite/uL; specificity of this diagnosis is 100%) and only patients diagnosed as infected were treated. Importation of malaria infections was present at a rate of 5 infections per 1,000 humans per year; these importations had a resistance allele frequency equal to the target frequency so helped stabilise allele frequencies against genetic drift during the burn-in. Parasite killing by both drugs was modelled using a mechanistic PK/PD framework with DHA modelled as 1-compartment dynamics and PPQ modelled as 2-compartment dynamics using dosing regimens and parameters as described previously in Kay & Hastings (2015).
- A twenty year “selection” phase. Resistance to PPQ was introduced at the end of the 10-year burn-in period by altering the drug sensitivity of the resistant allele (it previously had a sensitive profile to avoided being selected during burn-in). Importation was switched off to avoid it obscuring the selection process. Subsequent spread of resistance was monitored by recording its proportion of inoculations into the human host for each genotype every 5 days.

In OpenMalaria terminology the start of the burn-in is the start of the “intervention” and the selection phase is instigated by utilising a health-system change to redefine the drug sensitivity (IC50) of the resistance allele. In our work here, we use the “intervention” to introduce drug resistance alleles, switch off malaria importation, and monitor their subsequent spread. In this case DHA+PPQ was deployed throughout burn-in, and the only change made at ‘intervention’ was to introduce the resistant alleles. Similarly, if we had been investigating the impact of *hrp2*-deletions, RDTs would have been deployed during the entire burn-in period and the ‘selection’ would have used a health system change to introduce *hrp*2-deletions and track their spread.

Other assumptions in our OpenMalaria scenarios were as follows:

- The number of humans is a direct input into the simulation.
- Malaria prevalence is adjusted by altering the magnitude of the annual entomological inoculation rate.
- We modelled only perennial settings (i.e. no seasonality in malaria transmission although we relax this and allow seasonality when producing Figure S7).
- We did not include variation in PK or PD parameters so as to minimise variation between runs and focus on the interplay between selection coefficients, details of the regression windows, and population size.
- We assume there is no fitness cost associated with any of the alleles.

Selection coefficients were measured as described in the main text using the regression coefficients estimated using the base R package function lm.

Finally, note that the exact parametrisation is not important for the present purposes. The methodology was designed to be as simple and repeatable as possible because its primary purpose was simply to produce a series of advantageous alleles with differing selection coefficients to mimic a general selection process.

**SI.2 Incorporating de novo mutations into OpenMalaria**

We demonstrate the difficulty of incorporating de novo mutations in Figure 1 which calculates the dynamics of a mutation with a de novo rate of 10^-7^ per infection per generation.

An uncritical approach using small populations size to investigate very large populations would result in extremely large errors in observed rates of mutational input

Give these problems we then validated our algebraic approach described in the main manuscript by simulating the input of *de novo* mutations in OpenMalaria and checking our approach fitted the data. OpenMalaria is computationally expensive so we ran relatively small simulations of 100,000 individuals at 15% prevalence giving 15,000 malaria infections. We then used a relatively high mutation rate of 2x10^-5^ to achieve a reasonably rapid input of new de novo mutations (a mean wait time of around 22 years). Figure 6B of the main text show the mean times until mutational input obtained from OpenMalaria runs and the solid blue line shows the expected fit (Equation 6 of main text). This close fit between observational and theory serves to validate our approach as a mean of overcoming the computation limitations shown in Figure S6.

Finally, we include Figure 6A in the main text to show the dynamics of how mutation enter the populations and that most are lost by chance events; in this example from OpenMalaria there were at least 15 mutational inputs that were lost before one finally avoided stochastic extinction and became fixed in the population.

**2.1 Methodology.**

Mutation cannot currently be introduced directly in OpenMalaria but we can validate our methodology using importation to introduce new resistance mutations. This requires working around current limitations in OpenMalaria, the main ones being:

- It is not possible to change allele frequencies at an “intervention” (e.g. switch the malaria populations to be 100% drug sensitive), but it is possible to change the drug sensitivity of each allele
- Importation can be switched on/off as required at any time
- Importation, as currently coded, introduces new infections in the ratio of wildtype:resistant genotype used to start of the simulations.

We use importation of resistant infection to mimic the transmission of de novo resistnce mutations.

We therefore proceeded as follows:

- Phase 1. Warm up. Run the simulations for the usual 99 years with starting frequency of 50% resistance alleles. No selection occurs in this phase. is required to stabilise the simulations and ensure neither allele is lost by chance
- Phase 2: This is the first “intervention”. Switch the resistant alleles to be hyper-sensitive to the drug (IC50=0.1x normal so that selection removes them from the population leaving a 100% sensitive population at the end of Phase 2. Importation is required for the first 20 years of this phase to stabilise the epidemiology (set at 5 per 1000 persons per year) but the subsequent 80 years of selection against resistance removes them.
- Phase 3 This is the second “intervention”. The resistant alleles are switched back to their normal resistance phenotype (1.4x normal IC50 in the example investigated here). Importation is allowed which will introduce resistance mutations at the user-defined rate (see calibration below). Note that half the importations will be sensitive (importation reflects the original allele frequencies of 0.5 specified in Phase 1) but given the very low levels of importations this is unlikely to have much impact on epidemiology or on rate of spread of resistance alleles once they are established.

We track the introduction and spread (or loss) of new mutations using the OM output “nInfectByGenotype” which is defined in the wiki as “The number of human hosts with an infection (patent or not), for each genotype, at the time the survey is taken.”

An example is given on Figure 3 in this document. Note that when a new mutation arrives the value of nInfectByGenotype[resistant] changes from 0 to 1. Phase 3 can be analysed by recording all times at which nInfectByGenotype[resistant] are zero. The maximum time at which nInfectByGenotype[resistant] is zero therefore indicates the time of arrival of the first mutation that successfully establishes. These are the times recorded and plotted in Figure 2. We can also note the number of mutations which entered the population but were eventually lost as indicated by previous transitions in value from 0 to 1. The proportion of mutations that are lost, and how this varies with selection coefficient and epidemiology, is an interesting research question (Table 1 of Hastings 2004) which could not be explored here. Finally, note this method requires mutational input by importation to be sufficiently slow that only one mutational input is present at any one time (i.e. the ‘peaks’ in figure 3 are well separated). Note that in Figure 6A of the main text, the frequency of mutational input is around the maximum that can be handled in this IBM: the input at year 20 was lost at 24.7 years, but if it had survived longer at a few copies it would have been indistinguishable from the successful introduction at 24.8 years and the latter would have been erroneously interpreted as occurring at 20 years.

**2.2. Calibration for OM example**

We tracked 100,000 individuals at 15% prevalence giving 15,000 infections. Seasonality was absent. We used an IC50 increase for resistance alleles of x1.4 which gives s~0.05. This is consistent with field observations (see main text) was chosen as so that stochastic loss (i.e. failure to establish) is a significant effect.

Importation in OpenMalaria is described at <https://github.com/SwissTPH/openmalaria/wiki/ModelIntervMisc>

Which states “assuming an importation rate of r infections per person per time-step, for each simulated human and for each time-step the probability of that person receiving an imported infection is r”. Note the webpage then goes on to state

“The importation rate is actually specified with units of imported infections per 1000 people per year”

The number of de novo resistant mutations transmitted per year, r, is

r=Ni*ug [Equation S1]

where

Ni is number of malaria infections (i.e. infected people)

u is mutation rate per infection (i.e. per transmission)

g is the number of malaria generations per year)

We now use this number of mutant transmissions to calculate the required number of importations per 1,000 people. First divide number of mutant transmissions by human population size, N, to obtain the per-person importation rate. Then multiply by 1000 because OpenMalaria requires the number per 1000 people. Note that 50% of importations will be sensitive so need to double this importation rate to get correct importations of resistant mutations i.e.

Importation per 1,000 people per year =(Ni/N) u*g*1000*2 [Equation S2]

Noting that Ni/N is prevalence.

The observed rate, r’, i.e. the rate at which a mutation successfully enters and establishes itself in a population. Its calculation is analogous to equation S1 but with the addition of Ψ, the probability of a novel mutation surviving chance extinction (see discussion around Equation 4 in the main ms)

r’ =Ni u Ψg [Equation S3]

We calibrated OpenMalaria runs as follows:

- N= 100,000 individuals
- Ni=15,000 (because 15% prevalence)
- u: Mutation rate is 2e-5
- IC50 shift x1.4 leading to ’s’~0.05
- Ψ: probability of the mutations becoming established estimated at 0.025 (ie. s/2 with s~0.05).

These values gave

- Importation rate 0.036 (from Equations S2).
- Expected observed rate is 0.045 (from Equation 3 of the main text); this gives mean time to input of 1/0.045 = 22.2 years.

The estimate for a Ψ =0.05/2 =0.025 seems reasonable; see Table 1 in Hastings (2004). There appears to be a good fit between observed rate of input in OpenMalaria and expected rate; see Figure 6B of main text.

**SI.3. Additional plots.**

Figure S1. How choice of the day (after introduction of the advantageous allele) that regression starts affects estimated selection coefficients; regression lasted for 120 days (top row) or 720 days (bottom row). The left-hand column shows estimates of mean selection coefficient and the right-hand column shows coefficient of variation (CV) among these estimates. Methods: Mean and CV values were obtained from 100 simulations with different random seeds. Target frequency of resistance was set to 50%. Resistance levels corresponded to a 1.1-fold increase in sensitive IC_50_ value (blue crosses) and 1.4-fold increase in sensitive IC_50_ value (red crosses).


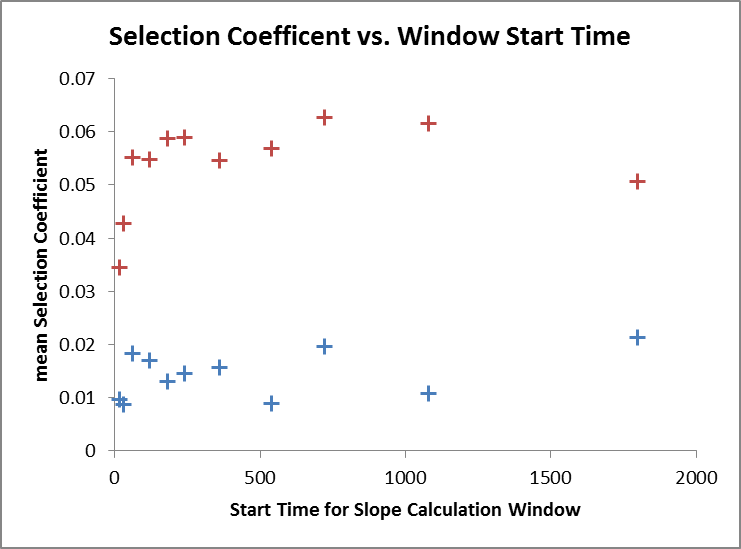

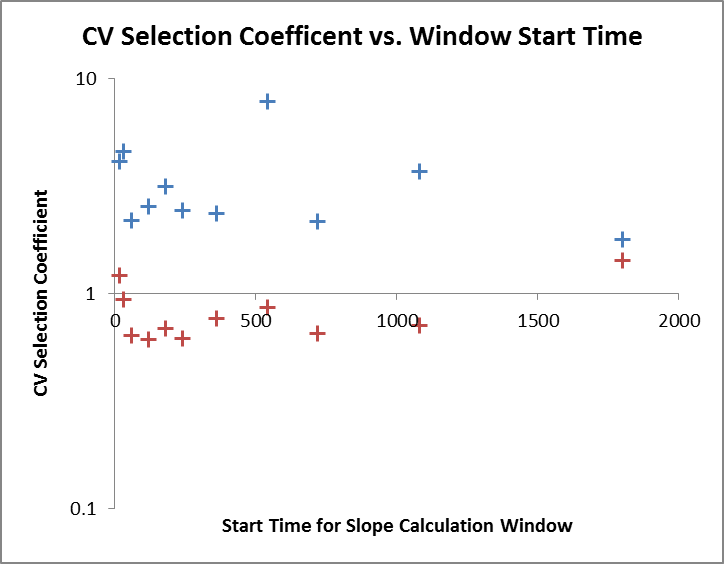


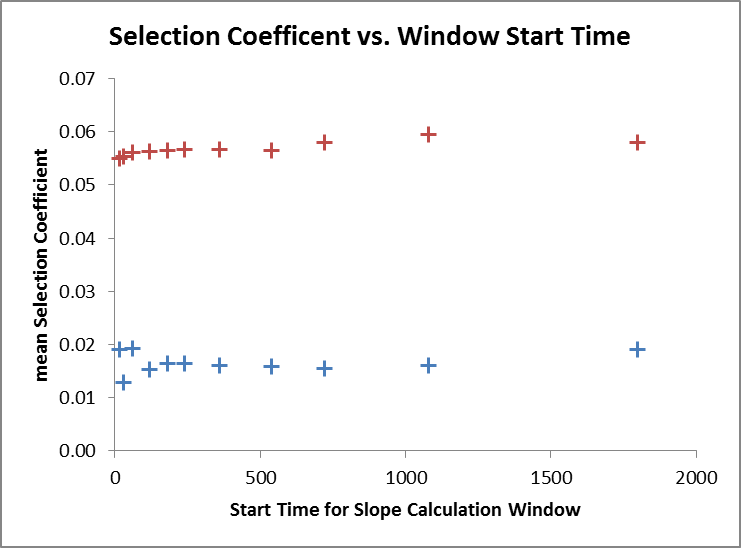

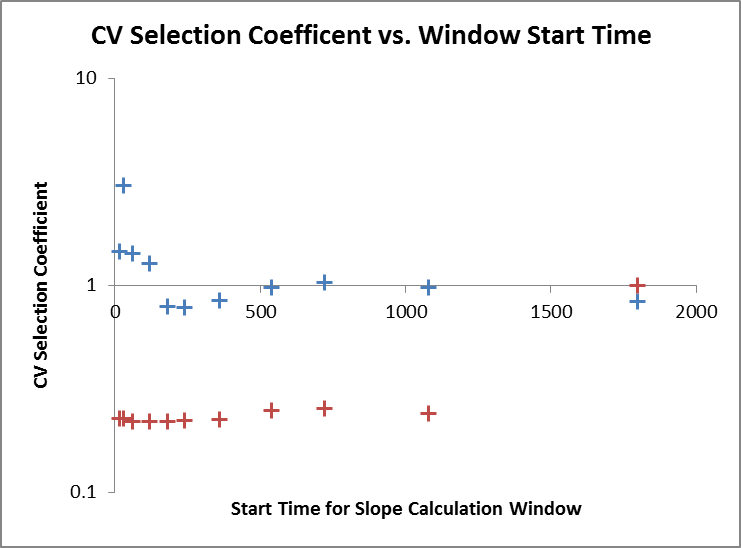


Figure S2. How duration of regression window affects estimated selection coefficients; regression started 15 days after introduction of the advantageous allele (top row) or after 60 days (bottom row). The left-hand column shows estimates of mean selection coefficient and the right-hand column shows coefficient of variation (CV) among individual estimates. Methods: Mean and CV values were obtained from 100 simulations with different random seeds. Target frequency of resistance was set to 50%. Resistance levels corresponded to a 1.1-fold increase in sensitive IC_50_ value (blue crosses) and 1.4-fold increase in sensitive IC_50_ value (red crosses).


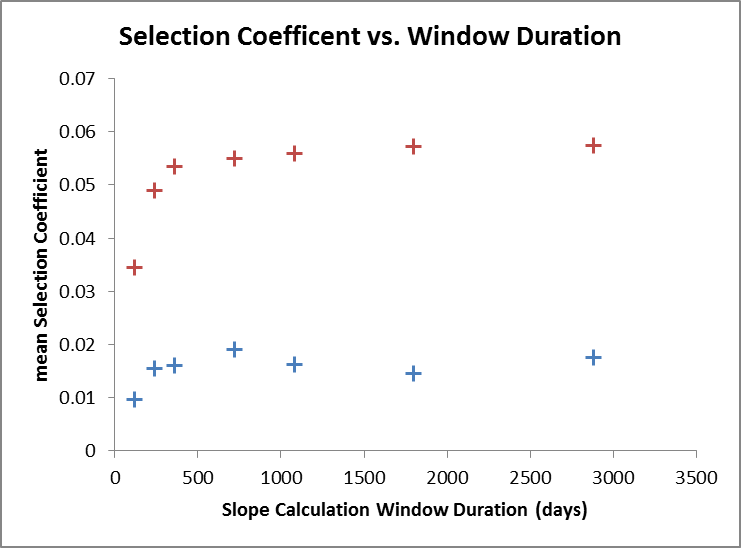

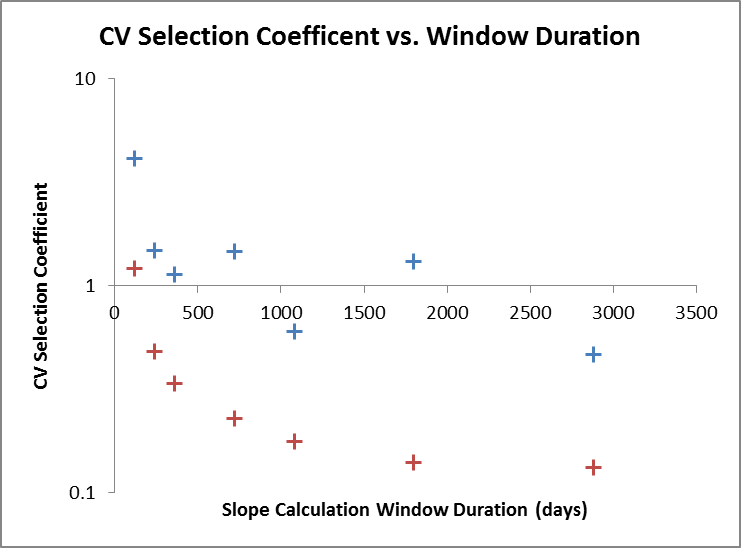


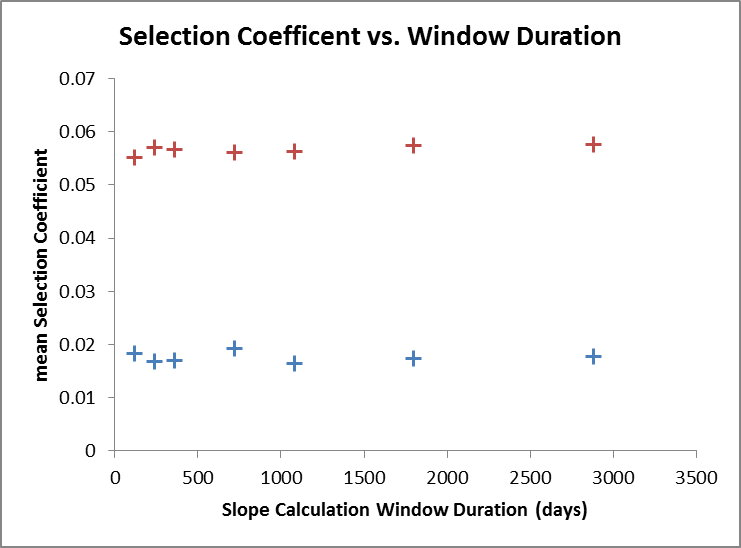

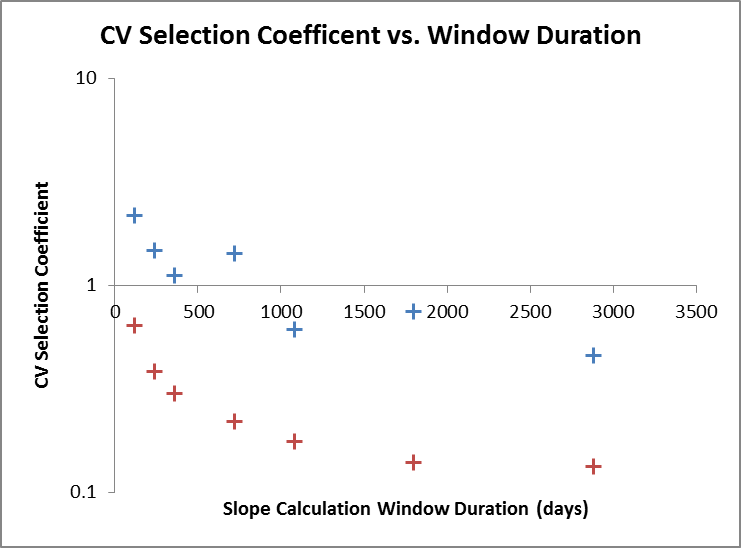


Figure S3. How frequency of resistance affects estimated selection coefficients; target frequency of resistance was set to 10%.

- Top row: as for figure S1, top row, but with 10% (cf 50%) target starting frequency of resistance.
- Second row: as for figure S1, bottom row, but with 10% (cf 50%) target starting frequency of resistance.
- Third row: as for figure S2, top row, but with 10% (cf 50%) target starting frequency of resistance.
- Fourth row: as for figure S2, bottom row, but with 10% (cf 50%) target starting frequency of resistance.

The left-hand column shows estimates of mean selection coefficient and the right-hand column shows coefficient of variation (CV) among individual estimates. Methods: Mean and CV values were obtained from 100 simulations with different random seeds. Resistance levels corresponded to a 1.1-fold increase in sensitive IC_50_ value (blue crosses) and 1.4-fold increase in sensitive IC_50_ value (red crosses).

Fig S3.


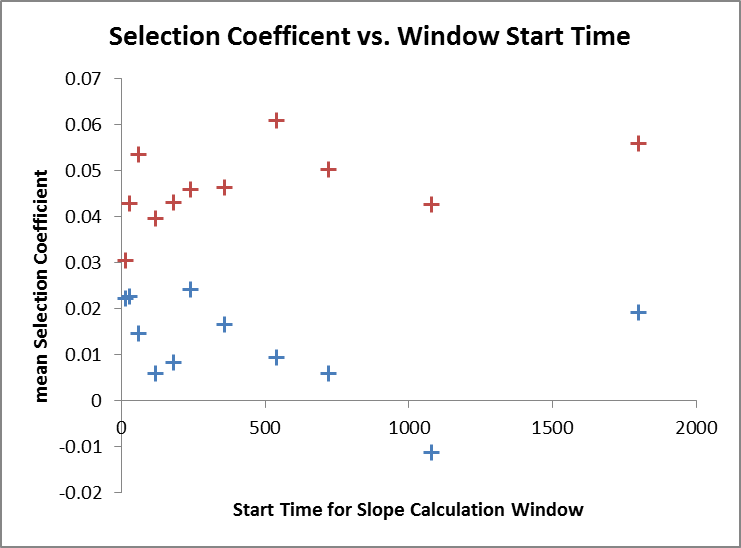

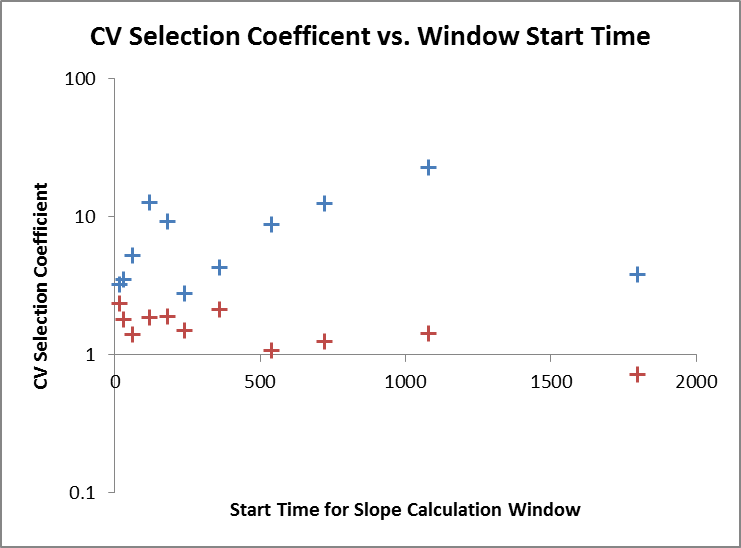


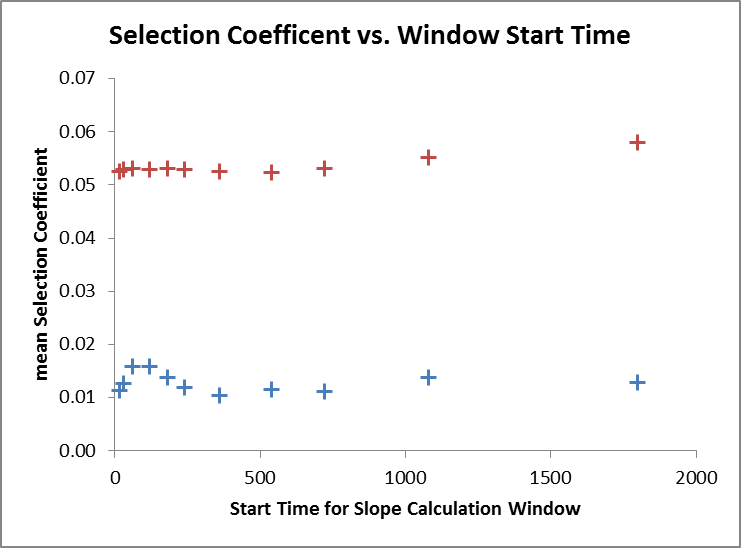

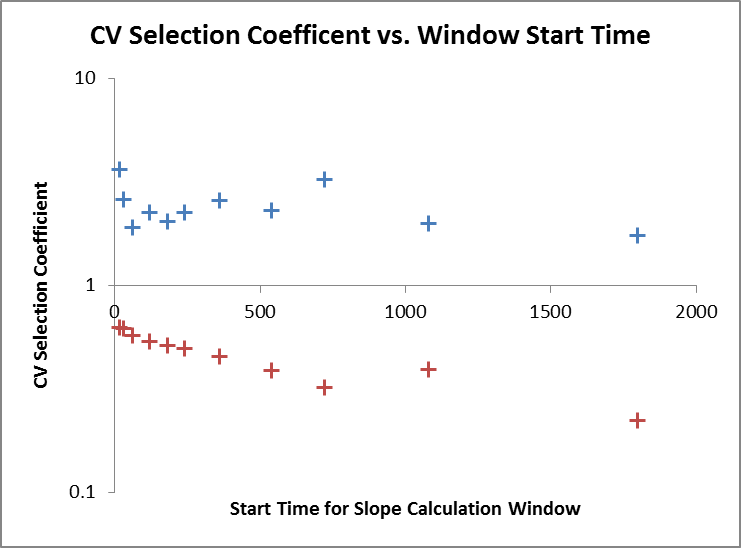


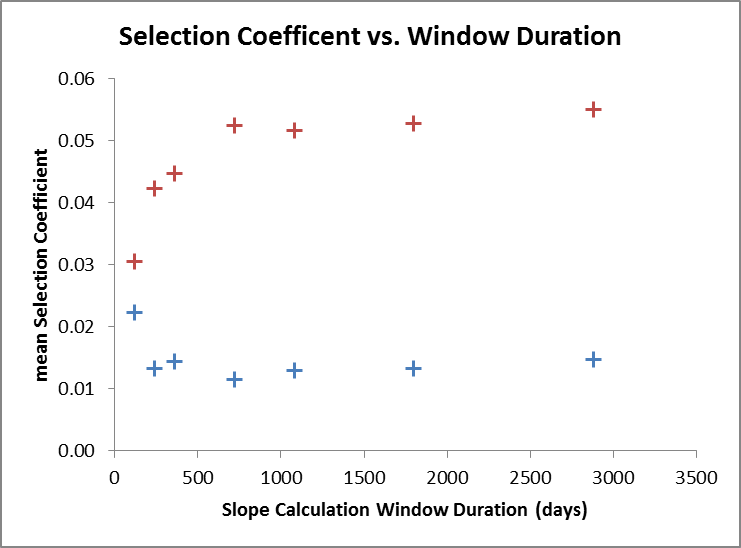

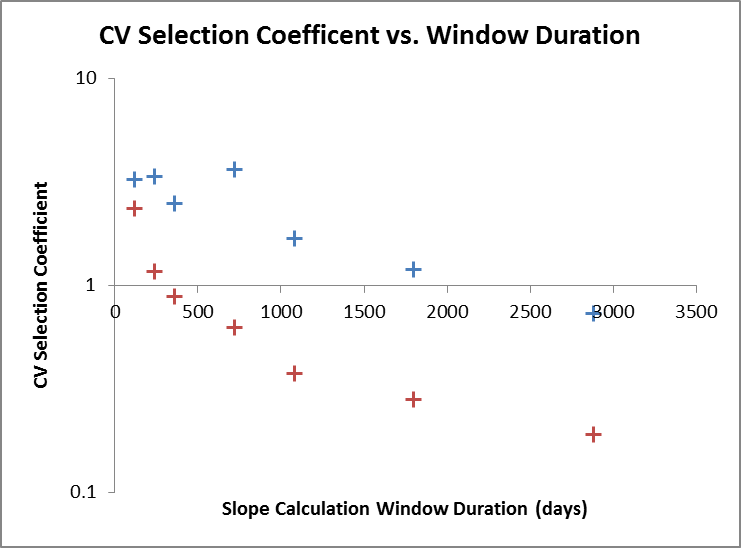


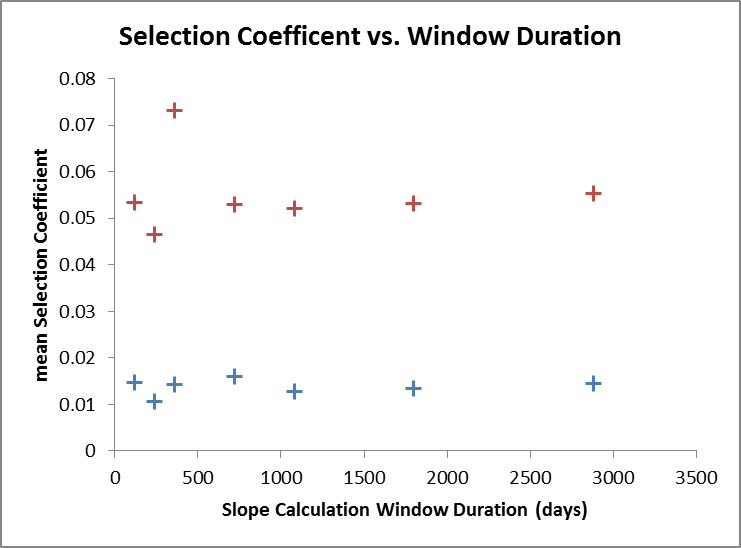

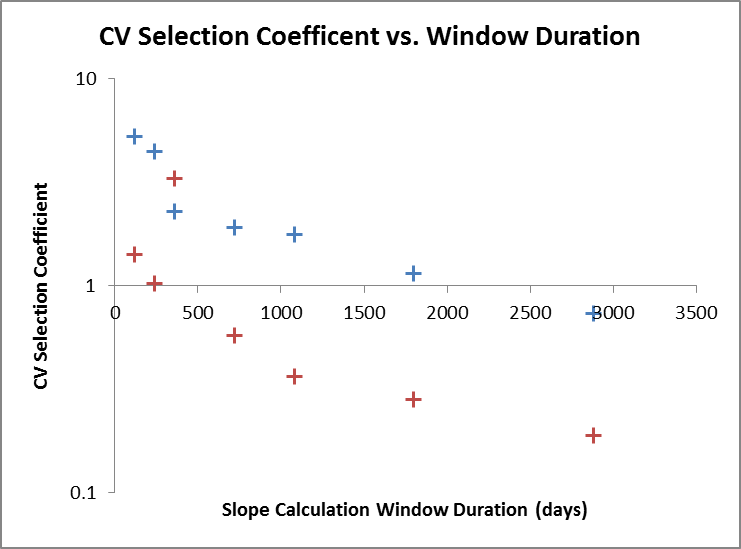


**Figure S4.** Median estimates of selection coefficient, s, plotted against regression window start time and duration; all medians come from 100 replicate runs. Panels A-C show median ‘s’ values obtained from IC_50_ shifts of 1.1x,1.4x, 2.4x respectively, with an initial advantageous allele frequency shift of 50%. Panel D shows median ‘s’ values obtained from an IC_50_ shift of 1.4x for an initial advantageous allele frequency of 10%.


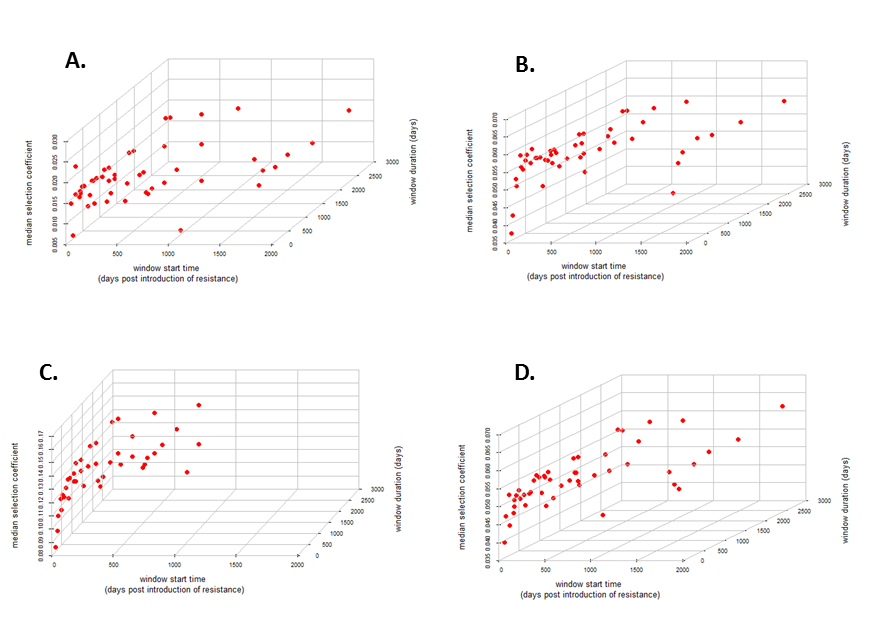


**Figure S5.** As from figure S4 but showing interquartile range of the ‘s’ values.


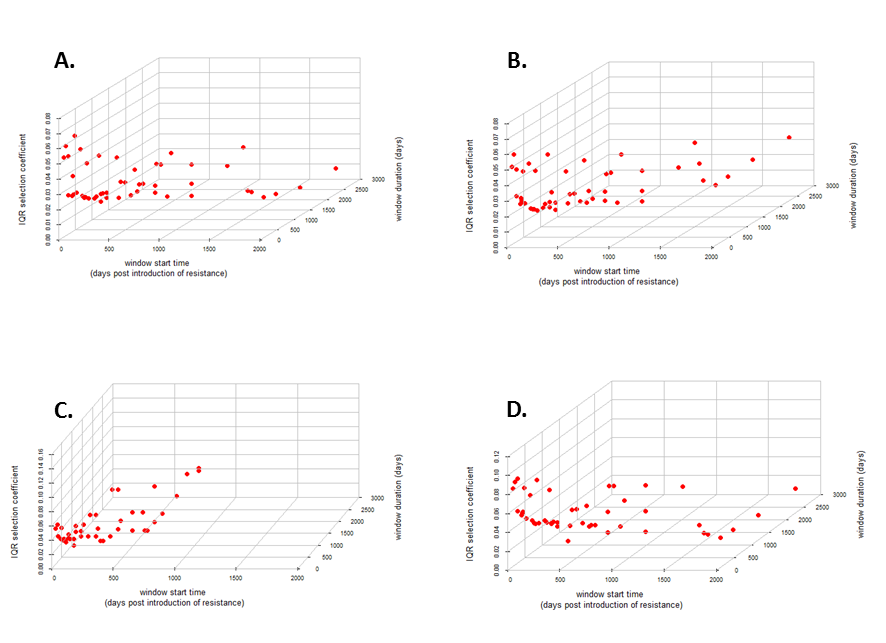


Figure S6. How simulated population size constrains the ability of IBMs to investigate the *de novo* input of mutations into pathogen populations. The red line is the mean waiting time until a mutation enters the population (Equation 6 of main text), the blue line is the time for that mutations to spread to detectable levels (assumed to be 1%) from its initial frequency of 1/population size (Equation 2 of main text) and the black line is expected total time for a mutation to enter the population and reach 1%. Calibration was as follows: mutation rate of 10^-7^; selection coefficient of 0.05; probability of a mutation surviving stochastic extinction of 0.05; 6 malaria generations per year.


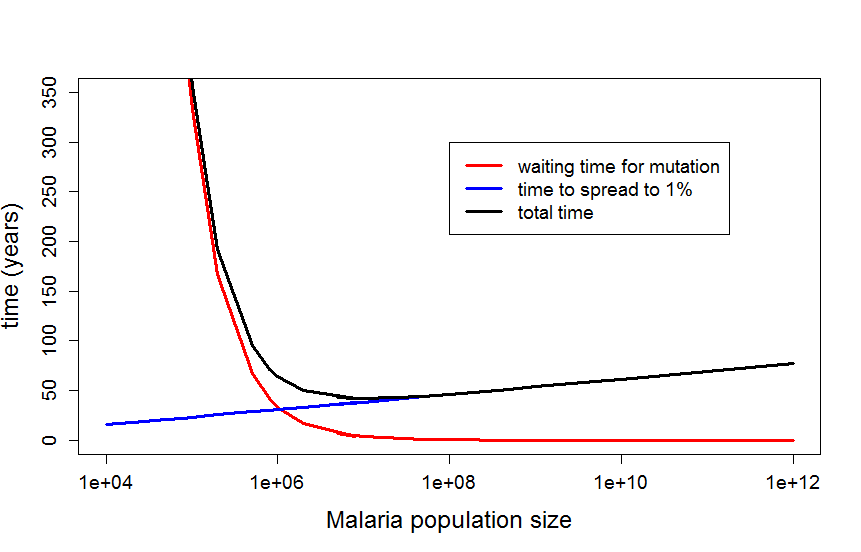


Figure S7. Output from OpenMalaria simulations showing their fit to theoretical expectations, particularly with respect to Figure 1 of the main manuscript. Six example simulations investigated drug treatment with dihydroartemisinic (DHA) plus piperaquine (PPQ) starting with an initial allele frequency of 1% (equivalent to -4.6 on the Y axis). The Y axis value equal to 90% allele frequency (i.e. ~2.2) is indicated by the red horizonal lines. The green lines show two simulations of neutral alleles (i.e. with no selective advantage): the dotted line is a simulation in an environment of seasonal variation in transmission, and the solid shows a simulation in the absence of seasonal variation. The light and dark grey lines show two simulations where seasonal variation in transmission occurs and the allele increases IC50 of PPQ by a factor of 1.4. The light and dark blue lines show simulations where seasonality is absent, and the alleles confer a 1.4-fold increase in PPQ IC50. All other factors are identical between the simulations. Panel (A) shows the full timescale, starting at year 10 when resistance is first introduced while panel (B) is an expanded region of panel A, focussing on the times at which allele frequencies lie between 50% (0 on the Yaxis) and 90%. Important points to note are

(i) Considerable random fluctuations in allele frequency occur at low initial frequencies. In fact, the (selected) light blue line initially falls in frequency before selection dominates and it starts to spread rapidly.

(ii) After these initial fluctuations, the four simulations of selected alleles are linear on the ln(R/S) scale, suggesting the absence of frequency-dependent selection.

(iii) One of the two neutral simulations (the one with seasonal variation) is rapidly lost through random fluctuations, while the other remains at roughly stable frequencies with longer-term random changes.

(iv) There is more temporally variation in frequency estimates in simulations where seasonality is present because OpenMalaria estimates frequency from transmissions every 5 days, and this results in small sample sizes, and hence large fluctuations, during periods of low transmission.

(v) Regression over the frequency period 50% to 90% gave estimates of selection coefficients of 0.05 and 0.05 for non-seasonal simulations and 0.06 and 0.05 for seasonal simulations, respectively. This suggests that, at least in this instance, seasonality has not significantly altered selection intensity for the drug-resistant alleles.

(A)


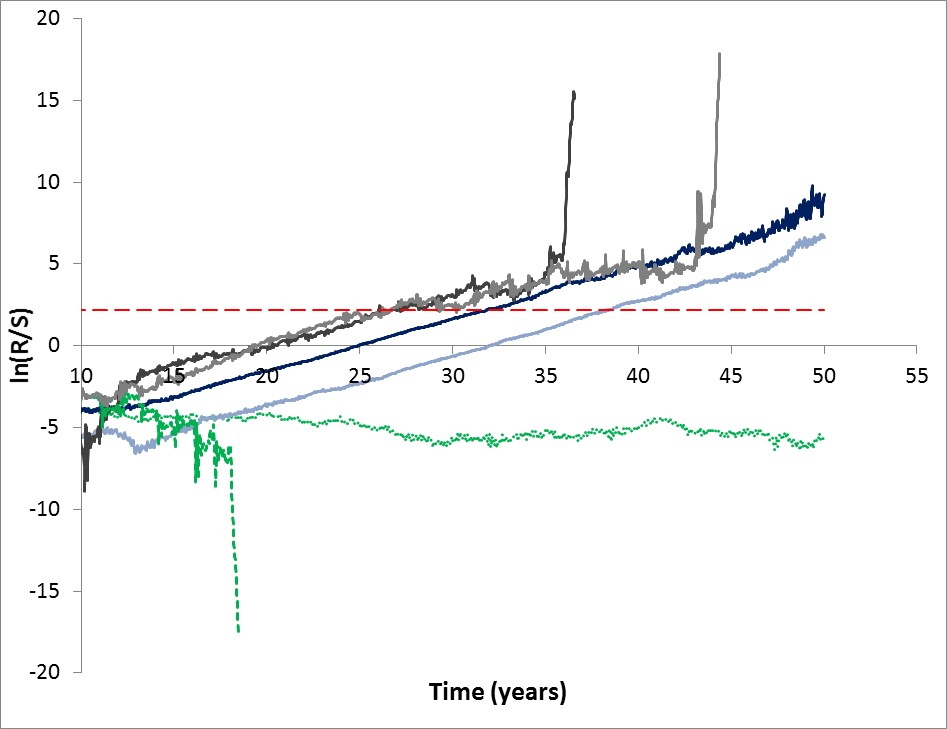


(B)


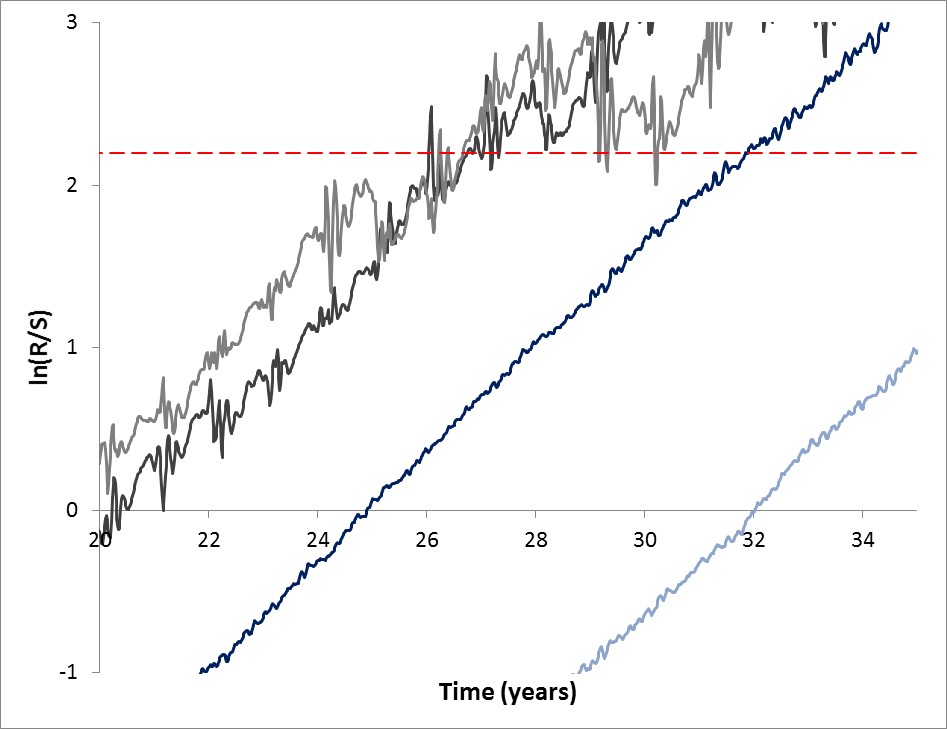


Citations.

Hastings, I. M. (2004). The origins of antimalarial drug resistance. *Trends in Parasitology, 20*(11), 512-518.

Kay, K., & Hastings, I. M. (2015). Measuring windows of selection for anti-malarial drug treatments. *Malaria Journal, 14*(1), 1-10. doi:10.1186/s12936-015-0810-4

Molineaux, L., Diebner, H. H., Eichner, M., Collins, W. E., Jeffery, G. M., & Dietz, K. (2001). Plasmodium falciparum parasitaemia described by a new mathematical model. *Parasitology, 122*(4), 379-391. doi:10.1017/S0031182001007533

Smith, T., Killeen, G., Maire, N., Ross, A., Molineaux, L., & Tediosi, F. (2006). Mathematical modeling of the impact of malaria vaccines on the clinical epidemiology and natural history of Plasmodium falciparum malaria: Overview. *American Journal of Tropical Medicine and Hygiene, 75*(2 Suppl), 1 - 10.

Smith, T., Maire, N., Ross, A., Penny, M., Chitnis, N., & Schapira, A. (2008). Towards a comprehensive simulation model of malaria epidemiology and control. *Parasitology, 135*(13), 1507 - 1516.

Smith, T., Ross, A., Maire, N., Chitnis, N., Studer, A., Hardy, D., . . . Tanner, M. (2012). Ensemble modeling of the likely public health impact of a pre-erythrocytic malaria vaccine. *PLOS Medicine, 9*(1).
